# Health Benefits of Screening for Co-occurring Alcohol-, Substance-, and Mood-related Conditions for At-Risk Populations: A Mathematical Modeling Study

**DOI:** 10.1007/s11606-026-10236-6

**Published:** 2026-02-25

**Authors:** Anna Bershteyn, Qinlian Zhou, Dyanna Charles, Mellesia Jeetoo, Maria R. Khan, Amy C. Justice, Natalie E. Chichetto, Brandon D. L Marshall, Adam J. Gordon, Stephen Crystal, Kendall J. Bryant, R. Scott Braithwaite

**Affiliations:** 1 Department of Population Health, NYU Grossman School of Medicine, 180 Madison Ave, New York, NY 10016 USA; 2 Center for Health Services Research, Rutgers University, New Brunswick, NJ USA; 3 Informatics, Decision-Enhancement, and Analytic Sciences (IDEAS) Center of Innovation, VA Salt Lake City Health Care System, Salt Lake City, UT USA; 4 Veterans Aging Cohort Study Coordinating Center, VA Connecticut Healthcare System, West Haven, CT USA; 5 Schools of Medicine and Public Health, Yale University, New Haven, CT USA; 6 Department of Epidemiology, University of Florida, Gainesville, FL USA; 7 Department of Epidemiology, Brown University School of Public Health, Providence, RI USA; 8 Department of Medicine, University of Utah, Salt Lake City, UT USA; 9 National Institute On Alcohol Abuse and Alcoholism, Bethesda, MD USA

**Keywords:** computer simulation, substance use disorder, mood disorders, health screening

## Abstract

**Background:**

Co-occurring alcohol, substance, and mood-related (CASM) conditions are prevalent, mutually reinforcing, and under-diagnosed contributors to morbidity, mortality, and health disparities.

**Objective:**

To evaluate screening strategies leveraging the predictive value arising from patterns of CASM co-occurrence in populations with high CASM prevalence.

**Design:**

Individual-based health risks model validated to predict US life expectancy and causes of death by sex and age decile, including CASM conditions of depression, anxiety, chronic pain, and unhealthy alcohol, tobacco, opioid and stimulant use. The model includes CASM co-occurrence patterns, mutual reinforcement across CASM conditions, and reduced engagement in other preventative care due to CASM.

**Participants:**

Veterans Aging Cohort Study (VACS), a large longitudinal cohort of in-care US veterans.

**Interventions:**

(1) Screening alcohol, tobacco, and/or depression symptoms; (2) adding further screening of CASM conditions likely to co-occur with those screened positive, with variation in the minimum co-occurrence rate; (3) screening all CASM conditions (hypothetical maximum).

**Main Measures:**

Estimated life expectancy (LE) and quality-adjusted life-years (QALYs).

**Key Results:**

The maximum strategy added 0.52 years to estimated LE (95% CI: 0.51 – 0.54) and 0.68 QALYs/person (95% CI: 0.67 – 0.69). Screening individual CASM conditions added a small fraction of this benefit, the largest LE gain from tobacco screening: 0.08 years (95% CI: 0.07 – 0.09). Screening for depression, alcohol, and tobacco provided 34.6% of the maximum strategy’s LE gain (0.19 years, 95% CI: 0.17 – 0.20). Additionally screening conditions with moderate (≥ 20%) probability of co-occurring with those already screened positive provided 84.8% of the maximum strategy’s LE gain. Screening all CASM conditions if depression, alcohol, and/or tobacco screened positive provided 86.6% of the maximum strategy’s LE gain.

**Conclusions:**

Compared to common practice of screening one or few CASM conditions, large health benefits are possible by further assessing CASM conditions most likely to co-occur with those already screening positive, improving health without increasing up-front screening burden in populations with high CASM prevalence.

**Supplementary Information:**

The online version contains supplementary material available at 10.1007/s11606-026-10236-6.

## BACKGROUND

Co-occurring alcohol, substance, and mood-related (CASM) conditions including depression, anxiety, chronic pain, and unhealthy alcohol, tobacco, opioid and stimulant use are prevalent in the United States (US)^1^ and other global settings.^2,3^ Their co-occurrence is partially explained by shared social, structural, and other predisposing factors – including social determinants of health such as poverty, discrimination, and history of trauma^4^ – as well as psychological mechanisms, such as dysregulation of reward response^5^ in the presence of allostatic load.^6^ CASM co-occurrence is further reinforced by causal relationships whereupon onset of one condition can trigger or exacerbate another.^7^ Conversely, remission or treatment of one CASM condition can ameliorate another.^8,9^ CASM remission or treatment also support other preventative health behaviors such as adherence to prescribed medications^10^ and meeting recommendations for diet and physical activity.^11^

Although CASM screening and treatment presents an important opportunity to lengthen life expectancy, health-related quality of life, and reduce health disparities, CASM conditions are under-diagnosed in the US and globally.^12^ The US Preventive Services Task Force (USPSTF) recommends screening for depression, anxiety, and unhealthy alcohol, tobacco, and other substance use in all US adults. Depression, alcohol, and tobacco use screening have been recommended for more than two decades^13^ and are more widely-practiced, though implementation remains variable. Comprehensive CASM screening is not commonly practiced in primary care settings,^14,15^ with providers citing concerns such as limited time in routine medical encounters^16,17^ and concerns about patient perceptions of large numbers of screens.^18,19^ Research is needed to inform efficient and effective CASM screening.

A potential innovation in CASM screening is leveraging the predictive value arising from CASM co-occurrence patterns to systematically screen additional conditions after one or more screen positive. The aim of this study is to explore the potential health benefits of such an approach in a population with high CASM prevalence: in this case, US military veterans in care at eight US Veteran’s Affairs (VA) health centers.^20^

## METHODS

### Mathematical Model Description

This study leveraged a previously developed, individual-based, Monte Carlo simulation of the competing risks of 19 mortality-causing conditions (Supplementary Table S1) that collectively account for 79% of all deaths in the US, and for > 70% of deaths by subgroup stratified by age decile and sex.^21,22^ Impacting the onset and remission of these mortality-causing conditions are 27 leading health risk factors in the US (Supplementary Table S2), including the seven CASM conditions that are the focus of this study: depression, anxiety, chronic pain, and unhealthy alcohol, tobacco, opioid, and stimulant use.

The model is described in detail in the Supplementary Methods and elsewhere.^21–23^ Briefly, the model simulates individuals from initialization until death in monthly increments. Each month, the model increments each simulated individual’s age, health-related risk factors, and conditions according to their age- and sex-specific rates of onset and remission.^22^ Active risks and conditions determine the quality-adjusted life-years (QALYs) accrued for the month, and active mortality-causing conditions are used to calculate the probability of death in that month. This process is repeated until death, at which time each individual’s lifespan, total QALYs, and cause of death are recorded.

### Data Sources

We adapted the model to match demographics, CASM prevalence, and CASM co-occurrence in the Veterans Aging Cohort Study (VACS), a large longitudinal cohort of in-care US veterans in Atlanta, Baltimore, Dallas, Houston, Los Angeles, New York, Pittsburgh, and Washington, D.C. This cohort is predominantly male (91.2%), with people living with HIV (PLHIV) comprising half of enrolled participants.^20^ We down-sampled PLHIV to constitute 0.7% of the total cohort, matching the approximate prevalence of HIV in male US adults, thus more closely reflecting a population treated primarily for general chronic medical conditions.^24^ PLHIV in this cohort were the focus of a prior analysis.^23^

Our simulations matched the proportion of the cohort with each of the 2^7^ = 128 possible combinations of the presence or absence of the seven CASM conditions ^9^ (Supplementary Table S3). The probabilities that remission of each CASM condition would lead to remission of each other CASM condition were previously derived for this cohort and are provided in Supplementary Table S4. These rates are referred to here as “spillover” of CASM treatment.

Sensitivity and specificity of screening tools for each CASM condition and effectiveness of treatment for each were obtained from literature review (Supplementary Table S5), taking into account diminution of effect between randomized controlled trials and real-world clinical practice. The odds ratios for adherence to preventative care upon successful treatment of each CASM condition were derived based on observed medication adherence in the cohort, and smaller odds ratios were used for preventative care requiring a lifestyle change, such as adherence to healthy diet and physical activity recommendations (Supplementary Table S5).^23^

### CASM Screening Simulations

We simulated a baseline scenario with no CASM screening, and a hypothetical maximum strategy of screening for all seven CASM conditions (depression, anxiety, chronic pain, and unhealthy alcohol, tobacco, opioid, and stimulant use) with referral to diagnostic assessment and treatment for conditions that screened positive (Supplementary Figure S1).

We then simulated strategies with a limited number of initial screens (depression only, alcohol only, tobacco only, depression + alcohol + tobacco, and depression + alcohol + tobacco + opioids + stimulants), with or without further screening of CASM conditions if a condition screens positive. In these scenarios, a screen was considered positive even if it was a false positive, given known limitations to the specificity of CASM screens (Fig. 2C).

Some strategies included a second round of screening, where additional conditions were screened if initial screens were positive, and if their co-occurrence probability (Fig. 2A) was above a pre-specified threshold. For example, with a stringent threshold of ≥ 80%, the only condition qualifying for additional screening would be anxiety after a positive depression screen, because the probability of anxiety co-occurring with depression in this cohort was 86%, while all other conditions co-occurred with < 80% probability. We examined a range of co-occurrence thresholds between 80% (very high probability of co-occurring) and 20% (moderate probability of co-occuring), in 10% increments.

### Assumed Effects of CASM Screening

CASM screening and treatment was incorporated into the model as shown in Supplementary Figure S3. If successfully treated, a CASM condition would: (1) be removed from person’s list of active risk factors, (2) potentially cause remission of other CASM conditions due to “spillover,” and (3) improve adherence to preventative care and healthy lifestyle recommendations (Supplementary Table S5).^23^ Estimates of the extent of “spillover” and the extent of improved adherence to preventive care were obtained from prior causal inference analyses using simulated randomized controlled trials constructed from the VACS cohort.^7–9,25–27^

### Main Outcomes

We reported the effect of CASM screening strategies on total life expectancy (chronological age at death) and on the remaining QALYs lived, compared to a counterfactual of no CASM screening. We further reported the percent change in the number of deaths observed for each of 10 leading causes: overdose, injury, cancer, lung disease, liver disease, cardiovascular disease, kidney disease, infectious diseases, and dementia. Due to the large number of CASM screening scenarios considered, we presented changes in causes of death from the counterfactual to one reference scenario of screening depression symptoms, unhealthy alcohol use, and tobacco use, followed by screening of conditions that have ≥ 50% co-occurrence with those that screened positive. In the Supplementary Appendix, we presented the cause-of-death analysis with an alternative reference scenario: screening all seven CASM conditions (depression, anxiety, chronic pain, and unhealthy alcohol, tobacco, opioid, and stimulant use).

### Sensitivity Analyses

We systematically assessed the sensitivity of results to uncertainty in model parameters. We varied one or multiple CASM-related model parameters across their uncertainty ranges (listed in Supplementary Tables 4 and 5), including uncertainty in CASM screen sensitivity and specificity, treatment success rates, and “spillover” rates.

In further sensitivity analysis, we disaggregated the health benefits of CASM screening according (1) direct alleviation of the screened condition, (2) “spillover” to other CASM conditions, and (3) improved adherence to preventative health recommendations. We also assessed the sensitivity of results to whether or not follow-on screening utilized standard screening instruments – i.e., having the same sensitivities and specificities as those used in initial screening (Fig. 2C). Because providers may choose to conduct more in-depth assessments once the first positive screen raises suspicion for co-occurring conditions, we tested an alternative assumption of 100% sensitivity and specificity of follow-on screening for co-occurring conditions.

## RESULTS

**Figure 1:**
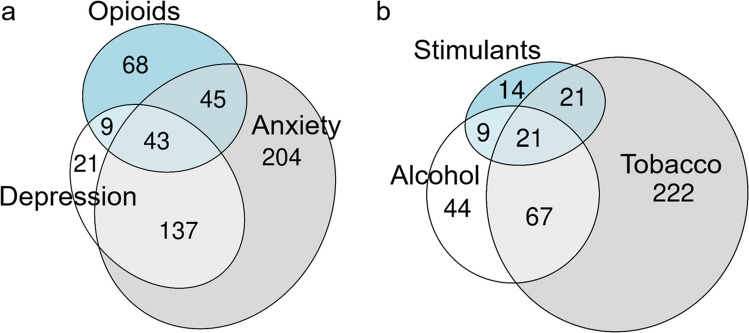
Euler diagrams showing co-occurring alcohol, substance, and mood (CASM) for illustrative triplets of conditions. Left diagrams shows the overlap of depression, anxiety, and disordered opioid use. Right diagram shows the overlap of disordered alcohol, tobacco, and stimulant use. Labels show the number of individuals in the study cohort with the indicated combinations of conditions. Although the figures only show up to three-way overlaps because larger numbers are difficult to visualize, the model mathematical incorporates up to seven-way overlaps, with 2^7^ = 128 possible combinations of CASM conditions.

CASM prevalence and co-occurrence were high in this cohort of urban US military veterans (Fig. 1), with 20.7% of individuals having two concurrent conditions, 18.1% having three concurrent conditions, and 14.0% having four or more concurrent conditions. Compared to what would have been expected if CASM conditions were distributed independently of one another (Supplementary Figure S2), the cohort had greater-than-expected prevalence of having no CASM conditions (21.2% observed vs. 10.2% expected) and greater-than expected prevalence of having four or more co-occurring conditions (14.0% observed vs. 6.9% expected), confirming the tendency of CASM conditions to cluster together.

**Figure 2:**
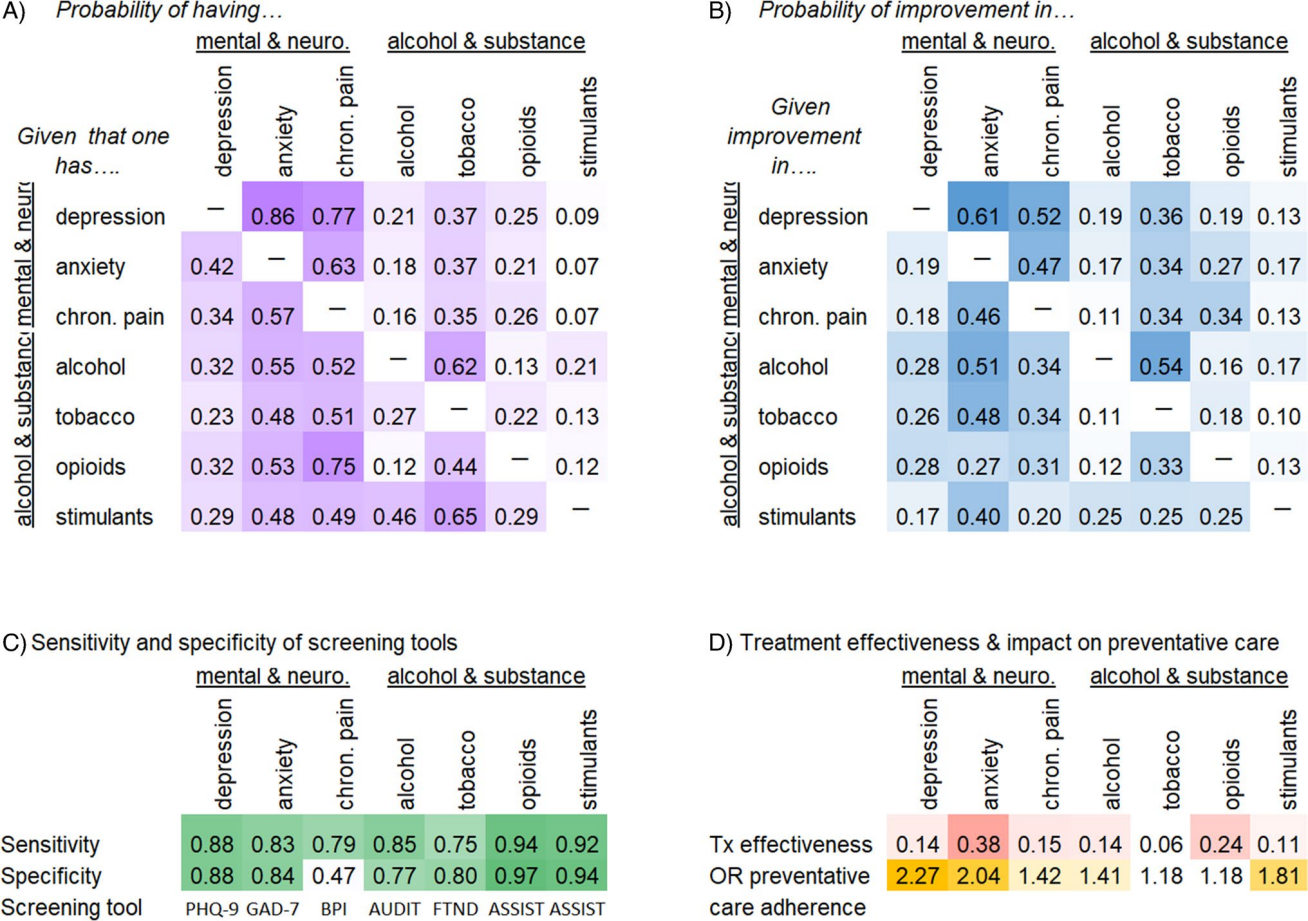
Heat maps showing the interactions of seven CASM conditions: (**A**) conditional probabilities of each condition co-occurring with each other condition, (**B**) causal effect of remission of each condition with remission of each other condition, (**C**) screen sensitivity and specificity for each condition, and (**D**) and treatment effectiveness, defined as probability of treatment success for each condition, and impact of successful treatment on adherence to chronic care such as maintenance medications reported as an odds ratio (OR). Uncertainty ranges and sources for these estimates are provided in Supplementary Table S5 and the Supplementary References. “Neuro” abbreviates “neurological.”

CASM co-occurrence was common within the mood disorder domain (e.g., 85.7% of those with depression had co-occurring anxiety), within the substance use domain (e.g., 64.6% of those using stimulant drugs use also used tobacco), and across domains (e.g., 75.1% of opioid users had chronic pain, 55.3% of those with unhealthy alcohol use had anxiety) (Fig. 2A). “Spillover” effects were generally strongest among conditions that co-occurred most often (e.g., 60.7% probability of remission in anxiety after successful treatment of depression). Exceptions to this pattern included: only 24.9% tobacco cessation after successful treatment of stimulant drug use disorder despite 64.6% co-occurrence; only 30.9% remission of chronic pain after successful treatment of opioid use disorder despite 75.1% co-occurrence (Fig. 2B and Supplementary Table S4).

**Figure 3:**
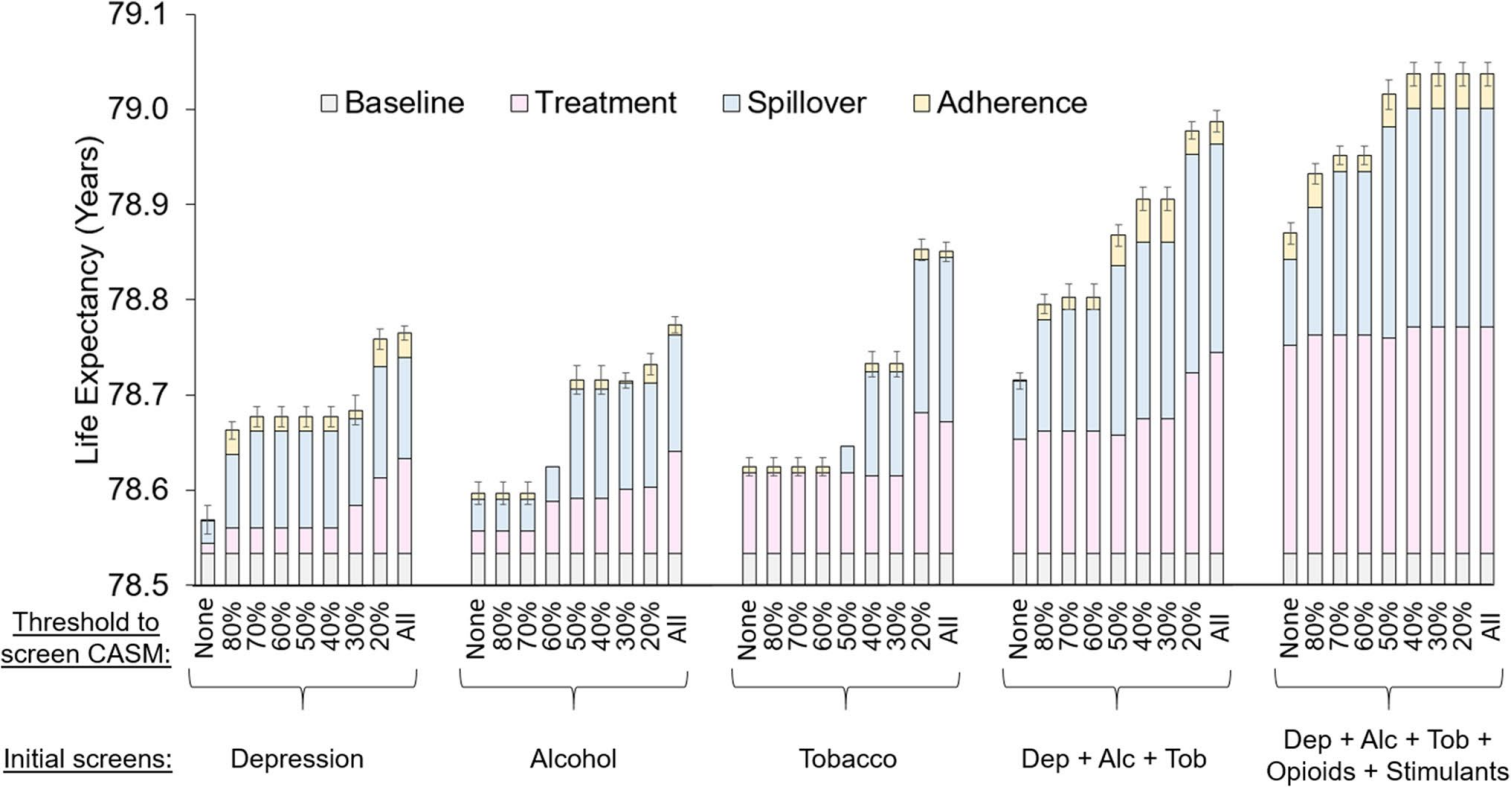
Impact on life expectancy of screening co-occurring alcohol, substance, and mood-related conditions (CASM). All strategies begin with screening an initial set of conditions recommended for US primary care, shown at the bottom of the figure. “None” denotes screening only these individual conditions without further screening of conditions that are likely to co-occur. “All” denotes screening all seven CASM conditions if any of the initially screened conditions screen positive. Percentages indicate the minimum probability of co-occurrence, above which another condition is screened if the initially screened condition screens positive. For example, at a threshold of 80%, only conditions with ≥ 80% probability of co-occurring with a positive condition are further screened, e.g., anxiety is screened if depression screens positive. Life expectancy gains are subdivided by mechanism into: (pink) direct effect of treating the screened conditions, (blue) “spillover” effect onto other CASM conditions due to causal associations in their remission, and (yellow) improved adherence to chronic care such as maintenance medications.

Baseline simulations of the cohort in the absence of any CASM screening estimated an average LE of 78.5 years (Fig. 3, grey bars) with 20.4 QALYs remaining from cohort initialization until death. A hypothetical maximum strategy of screening all CASM conditions increased LE by 0.52 years (95% CI: 0.51–0.54). When additionally considering quality of life, the maximum strategy added an average of 0.68 QALYs per individual (95% CI: 0.67–0.69).

Screening a single CASM condition added a small fraction of the hypothetical maximum benefit (Fig. 3). Of three individual screens analyzed (depression, alcohol, and tobacco), the smallest LE gain resulted from screening depression (0.04 years, 95% CI: 0.02–0.05 years) and the largest LE gain resulted from screening tobacco (0.08 years, 95% CI: 0.07–0.09 years), which provided 16.1% of the benefit of the maximum strategy. When incorporating quality of life (Supplementary Figure S3), the smallest gain resulted from screening alcohol (0.05 QALYs, CI: 0.04–0.06 QALYs), followed by depression (0.07 QALYs, CI: 0.06–0.09 QALYs) and tobacco (0.08 QALYs, CI: 0.07–0.09 QALYs), (Supplementary Table S5).

**Figure 4:**
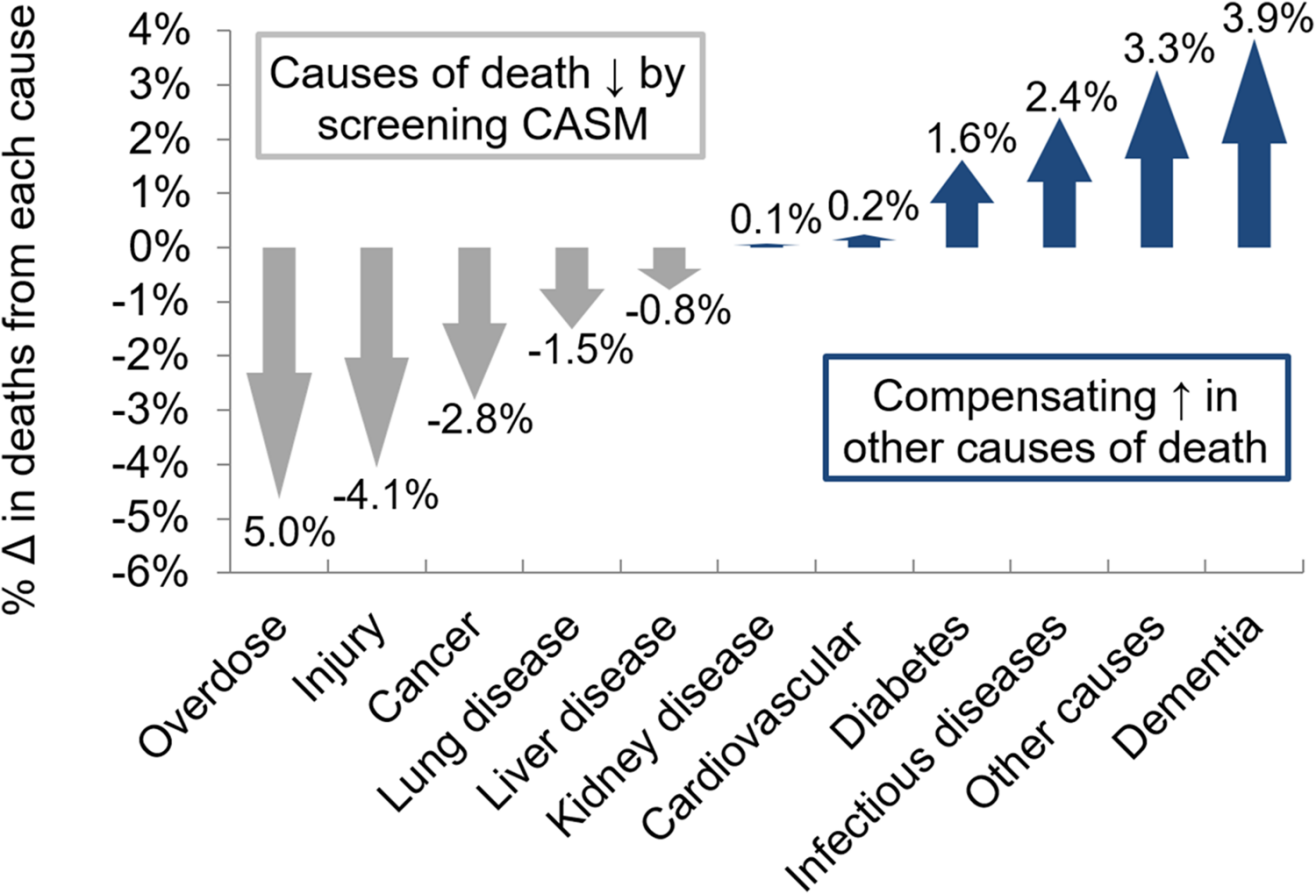
Effect of CASM screening on the distribution of causes of death. Arrows show the percent change in the number of deaths observed for each cause, comparing a counterfactual of no CASM screening to a reference strategy of screening depression symptoms, unhealthy alcohol use, and tobacco use, followed by screening of conditions that have ≥ 50% co-occurrence with those that screened positive. Categories have been simplified as follows: overdose combines overdoses from oral opioids, oral stimulants, and intravenous drugs. Injury combines accidental injury, homicide, and suicide. Cardiovascular combines heart diseases and stroke. Infectious diseases combines HIV, hepatitis, and respiratory illnesses. Dementia combines Alzheimer’s and Parkinson’s.

Mechanisms of LE and QALY gains differed according to the condition screened (colors of bars in Figs. 3 and 4). Tobacco screening primarily yielded health benefits through the direct health benefits of tobacco cessation (pink regions). Depression screening primarily yielded benefit through “spillover” effects that ameliorated other CASM conditions (blue regions). Alcohol screening also produced predominantly “spillover” effects but had a more sizable contribution of health benefits from improved adherence to other forms of preventative care (yellow regions).

Screening all three of depression, alcohol, and tobacco provided 34.6% of the maximum strategy’s LE gain (0.19 years, 95% CI: 0.17–0.20 years). Achieving ≥ 50% of the maximum strategy’s benefits required either screening a wider set of conditions initially – e.g., screening depression, alcohol, tobacco, opioid and stimulant – or adding further screening of conditions depending on which initial screens were positive. Screening all CASM conditions if tobacco use screened positive resulted in 60.5% of the maximum LE gain (Fig. 3) and 53.9% of the maximum QALY gain (Supplementary Figure S3). Screening all CASM conditions if depression, alcohol, and/or tobacco use screened positive resulted in 86.6% of the maximum LE gain and 83.1% of the maximum QALY gain.

We further explored how follow-on screening strategies could be informed by a priori knowledge of CASM co-occurrence rates in the population (Figs. 3 and 4). Achieving ≥ 50% of the maximum strategy’s benefit following an initial tobacco screen required screening any condition with ≥ 20% probability of co-occurring with tobacco use. In contrast, achieving ≥ 50% of the maximum LE gain after initially screening all three of depression, alcohol, and tobacco only required further screening conditions with ≥ 70% probability of co-occurring with those screened positive – a much higher threshold that in this cohort only required screening anxiety and chronic pain if depression screened positive.

**Figure 5:**
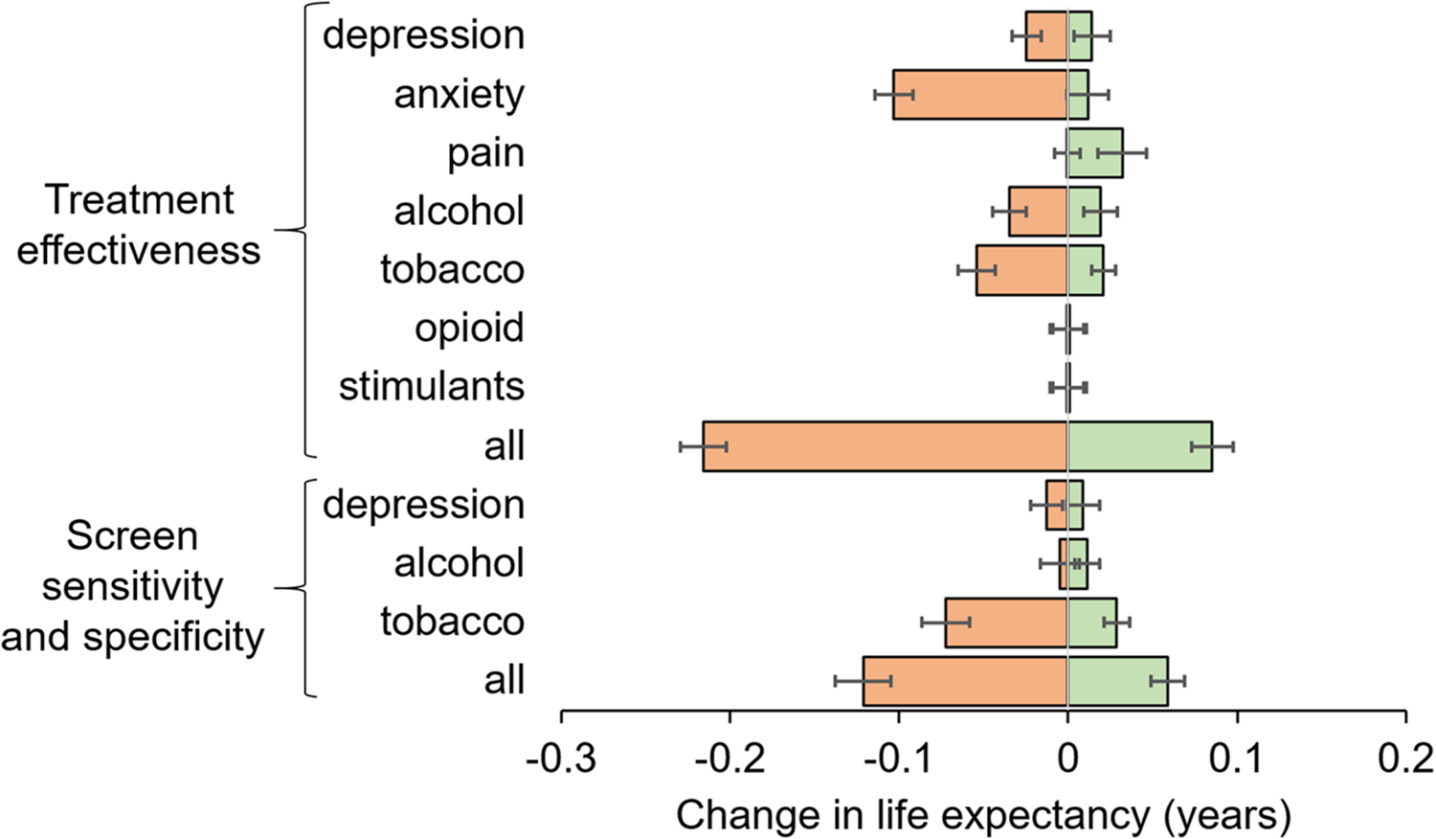
Tornado plot showing sensitivity of life expectancy improvements to uncertainty in model parameters governing screening and treatment. Uncertainty is shown relative to a reference scenario of screening depression symptoms, unhealthy alcohol use, and tobacco use, with further screening of conditions that have ≥ 50% probability of co-occurring with conditions that screened positive.

Projected deaths in the cohort were attributable to ten predominant causes, the largest two being cardiovascular disease and cancer (Supplementary Figure S4). CASM screening reduced some causes of death – particularly overdose, injuries, cancer, lung disease, and liver disease – with compensating increases in other causes such as dementia (Fig. 5). With the hypothetical maximum strategy, overdose deaths dropped by 27.4% and injury deaths by 7.1% (Supplementary Figure S5).

In sensitivity analysis, results were sensitive to uncertainty in treatment effectiveness – particularly if anxiety, tobacco, and/or alcohol treatment were less effective – and moderately sensitive to screen sensitivity and specificity, particularly for tobacco screening (Fig. 5). In further sensitivity analysis, if assessment of co-occurring conditions were to use a gold-standard diagnostic assessment rather than another screen, strategies with limited initial screening with broad assessment of co-occurring conditions would produce considerably larger health benefits (Supplementary Figure S6). Screening all CASM conditions in those who screen positive for only depression, only alcohol, or only tobacco would achieve similar impact to the maximum strategy. Achieving ≥ 50% of the maximum LE gain (+ 0.26 life-years) could be accomplished through diagnostic assessment of conditions with ≥ 47.3% co-occurrence with an initial tobacco screen, ≥ 55.1% co-occurrence with an initial alcohol screen, or ≥ 30.5% co-occurrence with an initial depression screen.

## DISCUSSION

We estimated LE and QALY gains from CASM screening, with or without an approach whereby knowledge of the population’s rates of CASM co-occurrence inform follow-on screening. A hypothetical maximum strategy of screening all CASM conditions could extend LE by more than half a year, even after accounting for low CASM treatment success rates. Several less intensive screening strategies could exceed one-quarter of a year in LE gains. These included screening for depression, alcohol, and tobacco plus further screening of conditions that co-occur very often (≥ 70% probability) with those that screened positive; or screening only one condition initially (depression, alcohol, or tobacco) with further screening of conditions that co-occur relatively often (≥ 30–50% probability) if the initial screen is positive.

Reported LE gains reported were on par with some of the most strongly recommended preventative care screenings in the US. Breast cancer screening in women ages 40 and older is estimated to add 0.40 years to LE on average;^28^ colorectal cancer screening in adults ages 45–75 is estimated to add 0.36 years;^29^ and cervical cancer screening in women ages 21 and older is estimated to add 0.27 years.^30^ Impacts were also on par with preventative screening and treatment for specific conditions concentrated in high-risk populations. For example, hepatitis C virus screening and treatment in people with a history of injection drug use is estimated to add 0.23 years to LE.^31^ Our study takes into account the low treatment success rates observed for CASM conditions (see Supplementary References S106–S107), but nevertheless estimates comparable health benefits to other treatable and preventable health conditions. This was in part due to ‘spillover’ benefits, in which successfully treating one CASM condition can induce remission of other CASM conditions. Results were sensitive to treatment effectiveness, suggesting that more effective treatments could greatly enhance health benefits of CASM screening.

Generalizability of our findings will depend on different populations’ CASM prevalence, co-occurrence rates, baseline patterns and disparities in the number of CASM conditions screened, and other co-occurring health conditions impacted by CASM. In a previous study, we evaluated CASM screening among HIV-positive US adults, for whom CASM is particularly deleterious due to the health harms of disrupting adherence to daily antiviral medications.^32^ We found that nearly a full year of LE can be gained by screening a small set of CASM conditions initially, followed by screening for other CASM conditions if any initial screens are positive.^23^ The current study focuses on military veterans, who have disproportionate exposure to trauma,^33^ a predisposing factor for CASM,^4^ and sometimes access care through the VA because of its “safety net” role,^34^ making the cohort more inclusive of individuals with socioeconomic disparities – another CASM risk factor.^35^ Notably, social and structural factors that perpetuate CASM in health disparity populations are known to act with CASM to disrupt effective care and prevention of other health conditions.^10,11^ Expanded CASM screening and treatment could be one component of breaking the cycles that perpetuate health disparities in the US^36^ and globally.^37^ Additional discussion of the study’s implications can be found in the  Supplementary Discussion.

Our study has several important limitations. Generalizability was limited by reliance on cohort data from US military veterans who were predominantly male, over age 30, experienced high rates of specific conditions such as post-traumatic stress disorder, and had access to unique healthcare resources at the VA. Our study did not incorporate financial, time, and other constraints, and was limited to only seven CASM conditions, each represented monolithically (e.g., cocaine use was bundled into a monolithic stimulant use disorder; prescription opioid misuse was bundled with opioid use disorder). Our scenarios focused on alternative screening strategies and did not vary screening instruments, referral mechanisms, or diagnostic and treatment methods. We relied on limited evidence, especially for “spillover” effects, necessitating extensive sensitivity analyses. Finally, like all modelling studies, we made numerous simplifications, including neglecting correlation of screen sensitivities and specificities and time delays between screening, diagnosis, treatment, and remission. In-depth discussion of each limitation and its implications can be found in the Supplementary Discussion.

In this simulation study, scenarios adding CASM screening for individuals with high probabilities of CASM co-occurrence, under favorable conditions for referral and treatment access, showed promise in providing substantial health benefits with relatively small increments in screening burden. Empirical studies are needed to confirm predicted benefits in populations with high CASM prevalence.

## Supplementary Information

Below is the link to the electronic supplementary material.

Supplementary file1 (DOCX 957 KB)
