# Supplementary material for: Health Benefits of Screening for Co-occurring Alcohol-, Substance-, and Mood-related Conditions for At-Risk Populations: A Mathematical Modeling Study
Source: J Gen Intern Med. 2026 Feb 25;41(9):2456–64. doi: 10.1007/s11606-026-10236-6 (PMC13304428; doi:10.1007/s11606-026-10236-6)
Supplement: Supplementary file 1 — Supplementary file1 (DOCX 957 KB) [file 11606_2026_10236_MOESM1_ESM.docx]

**Supplementary Appendix to: Health benefits of screening for co-occurring alcohol-, substance-, and mood-related conditions for at-risk populations: a mathematical modeling study**

Anna Bershteyn, PhD^1*^

Qinlian Zhou, PhD^1^

Dyanna Charles, MPH^1^

Mellesia Jeetoo, MPH^1^

Maria R. Khan, PhD, MPH^1^

Amy C. Justice, MD, PhD^4,5^

Natalie E. Chichetto, PhD, MSW^6^

Brandon D.L. Marshall, PhD^7^

Adam J. Gordon, MD MPH^3,8^

Stephen Crystal, PhD^9^

Kendall J. Bryant, PhD^10^

R. Scott Braithwaite, MD, MSc^1^

^1^ Department of Population Health, NYU School of Medicine, New York, NY

^2^ Center for Health Services Research, Rutgers University, New Brunswick, NJ

^3^ Informatics, Decision-Enhancement, and Analytic Sciences (IDEAS) Center of Innovation, VA Salt Lake City Health Care System, Salt Lake City, UT

^4^Veterans Aging Cohort Study Coordinating Center, VA Connecticut Healthcare System, West Haven, CT

^5^Schools of Medicine and Public Health, Yale University, New Haven, CT

^6^Department of Epidemiology, University of Florida, Gainesville, FL

^7^ Department of Epidemiology, Brown University School of Public Health, Providence, RI

^8^Department of Medicine, University of Utah, Salt Lake City, UT

^9^Center for Health Services Research, Rutgers University, New Brunswick, NJ^7^

^10^National Institute on Alcohol Abuse and Alcoholism, Bethesda, MD

*Correspondence to: [Anna.Bershteyn@nyulangone.org](mailto:Anna.Bershteyn@nyulangone.org), 180 Madison Ave, New York, NY 10016, USA.

**Supplementary Methods**

We leverage a previously developed individual-based Monte Carlo model that simulates individuals, one at a time, from initialization until death. The simulation records person-time lived with different health conditions that are potential causes of death, as well as risk factors that do not directly cause death, but that increase the risk of developing conditions that could cause death, and of developing other risk factors. Risk factors and conditions included in the model are:

| **Supplementary Table S2: Health risk factors** |
| --- |
| Depression |
| Anxiety Tobacco use |
| Chronic pain use |
| Unhealthy alcohol use |
| Tobacco use |
| Opioid use |
| Stimulant use |
| Bipolar disorder |
| Cardiovascular disease |
| Diabetes |
| F.H.* Alzheimer's |
| F.H.* diabetes |
| F.H.* hypertension |
| F.H.* Parkinson's |
| Head trauma |
| High cholesterol |
| HIV infection |
| HPV infection |
| Hypertension |
| Immunocompromised |
| Malnutrition |
| Obesity |
| Poor air quality |
| Poverty |
| Sedentary lifestyle |
| Unvaccinated |
| Viral hepatitis |
| *F.H.: Family history |

| **Supplementary Table S1: Mortality-causing conditions** |
| --- |
| Accidental injuries |
| Alzheimer’s |
| Cerebrovascular disease (stroke) |
| Cancer: breast |
| Cancer: cervical |
| Cancer: colorectal |
| Cancer: lung |
| Cancer: others |
| Cancer: prostate |
| Diabetes |
| Heart disease |
| HIV/AIDS |
| Homicide |
| Kidney disease |
| Liver disease |
| Lung disease |
| Drug overdose |
| Parkinson’s disease |
| Pregnancy and childbirth |
| Respiratory infections |
| Suicide |

CASM

At initialization, each individual is assigned an age, sex, and CASM status representative of the study cohort at the time of data analysis. CASM status is assigned in order to incorporate both the overall prevalence of each CASM condition in the cohort, and its probability of co-occurring with every other CASM conditions. Initial states are selected to match the combinations of CASM conditions observed in the study cohort, assigning each of the 2^7^=128 possible combinations of CASM conditions as illustrated below:

**Supplementary Table S3. Combinations of CASM conditions.**

|  | *Depression* | *Anxiety* | *Pail* |  | *Alcohol* | *Tobacco* | *Opioid* | *Stimulant* |
| --- | --- | --- | --- | --- | --- | --- | --- | --- |
| *Combination 1* | No | No | No |  | No | No | No | No |
| *Combination 2* | **Yes** | No | No |  | No | No | No | No |
| *Combination 3* | No | **Yes** | No |  | No | No | No | No |
| *Combination 4* | No | No | **Yes** |  | No | No | No | No |
| *Combination 5* | No | No | No |  | **Yes** | No | No | No |
| *Combination 6* | No | No | No |  | No | **Yes** | No | No |
| *Combination 7* | No | No | No |  | No | No | **Yes** | No |
| *Combination 8* | No | No | No |  | No | No | No | **Yes** |
| *Combination 9* | **Yes** | **Yes** | No |  | No | No | No | No |
| *Combination 10* | **Yes** | No | **Yes** |  | No | No | No | No |
| *Combination 11* | **Yes** | No | No |  | **Yes** | No | No | No |
| *Combination 12* | **Yes** | No | No |  | No | **Yes** | No | No |
| *…* | … | … | … |  | … | … | … | … |
| *Combination 128* | **Yes** | **Yes** | **Yes** |  | **Yes** | **Yes** | **Yes** | **Yes** |

In scenarios involving CASM screening, individuals who possess the CASM conditions that are included in screening have a probability of screening positive, dictated by each condition’s sensitivity (Supplementary Table S2). If a condition screens positive, further screening may be conducted if a condition screened positive and a strategy involves screening closely-associated conditions. For example, in this cohort, 86% of individuals with depression having co-occurring anxiety. If an individual with Combination 9 above (depression and anxiety) were to screen positive for depression, and the threshold for further screening is a co-occurrence probability of 86% or below, then they would receive anxiety screening. Conditions that screen positive are assumed to receive diagnostic assessment and treatment, with the assumption that treatment effectiveness (rather than efficacy) is relatively low (Supplementary Table S2) due to challenges patients face in accessing and benefitting from treatment. If a condition is successfully treated, it may trigger remission of additional CASM conditions based on the observed causal “spillover” effect across CASM conditions (Supplementary Table S4). The process of screening CASM conditions, applying treatment, and incorporating “spillover” is shown in Supplementary Figure S3.

To model the effect of CASM screening and treatment on life expectancy and quality-adjusted life-years (QALYs), individuals are simulated forward in time, one at a time, until death. The model advances in monthly timesteps. Each each timestep begins by incrementing the individual’s age by one month. Then, risk factors are updated by applying an age/sex-specific incidence rate of new risk factors, or resolution of currently active risk factors. Next, a similar procedure is applied to update the presence or absence of mortality-causing conditions. Finally, each mortality-causing condition is checked to determine whether the individual has died on that timestep. If they have died, then their lifespan, quality-adjusted life-years (QALYs), and cause of death are recorded. Otherwise, their age is incremented by an additional 1 month and the cycle repeats. The process is illustrated below:


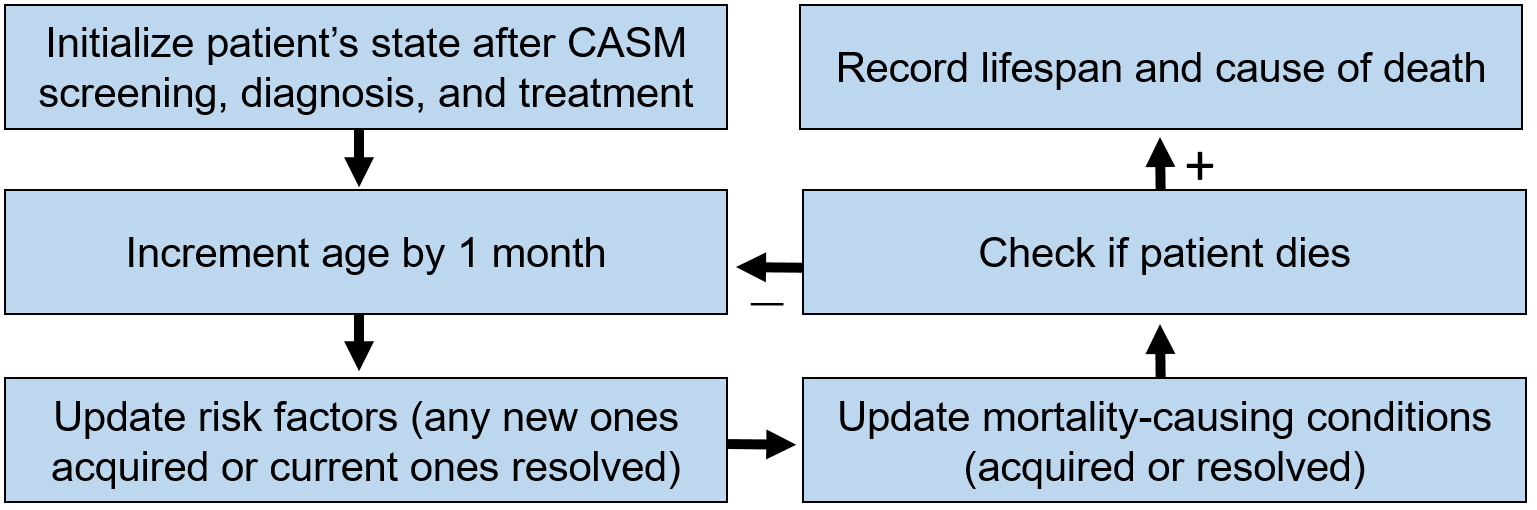


Updates to risk factors are conducted by applying each risk factor’s age/sex-specific incidence rate and resolution rate. The monthly age/sex-specific incidence rate of the $i^{th}$ risk factor, denoted $I_{i}$, is converted into a probability $P_{i}=1-e^{I_{i}}$ that the risk factor is acquired on that timestep. This is applied if the individual does not already have the risk factor. The monthly age/sex-specific resolution rate $R_{i}$ and is converted into a probability $P_{i}=1-e^{R_{i}}$ that the risk factor resolves on that timestep. This is applied if the individual already has the risk factor.

The model then updates the incidence and resolution rates of all risk factors and mortality-causing conditions according to the new set of risk factors present. For example, suppose an individual does not currently have the $j^{th}$ mortality-causing condition, the incidence of which depends on the presence or absence of the $i^{th}$ risk factor. If the $i^{th}$ risk factor was acquired on this timestep, then the incidence of $j^{th}$ condition is multiplied by the rate ratio (RR) for $j^{th}$ condition to develop on the presence of the $i^{th}$ risk factor:

$$I_{j\_ new}={RR}_{ij}\times I_{j\_old}$$

If multiple risk factors are acquired or resolved in the same timestep, their RRs are multiplied together. The updated probability of incidence/resolution of conditions is then applied in the same manner as the risk factors, e.g., $P_{j}=1-e^{I_{j}}$. The effect of risk factors on the incidence of other risk factors is applied in the next timestep.

While the above calculations were configured to reflect the average population-wide changes in the prevalence of conditions and risk factors as populations age, a limitation is that they do not capture the shorter time-scales of on-and-off patterns of some risk factors and conditions, for example, episodic depression.

The model determines whether the individual has died on the current timestep of any currently active mortality-causing conditions. Conditions are simulated as causing either rapid demise (e.g., accidental injuries, homicide, suicide) or long-term disease leading to demise (e.g., Alzheimer’s, diabetes, HIV, hypertension, Parkinson’s, cancers). Some conditions can cause both rapid demise or long-term disease leading to demise (e.g., chronic liver disease, cerebrovascular disease, heart disease). The long-term component of the risk of death for these diseases can be modified by (1) the effectiveness of treatment, and (2) the adherence to treatment.

The model tracks total survival time, time lived with each risk factor and mortality-causing condition, and the ultimate cause of death for each individual. By multiplying the utility weight for each month of life lived with conditions (Supplementary Table S5), the model reports quality-adjusted life-years lived, in addition to total life-expectancy and cause-of-death distribution for the cohort as its primary outputs.

The model represents a simplified process of CASM screening, diagnostic assessment, treatment, and the effects of successful treatment on other CASM and non-CASM conditions (Supplementary Figure S1).

We assumed an idealized (100%) sensitivity and specificity of diagnostic assessments because these are generally the “gold standard” against which performance is evaluated. However, diagnostic assessments can be fallible, possibly leading to slight over-prediction of effect sizes. Uncertainty in the quality of diagnostic assessment could be considered as one contributor to the very wide range of treatment effectiveness that we explored in sensitivity analysis.

Individuals with an underlying CASM condition who screen positive were assumed to receive referral to diagnostic assessment and linkage to treatment. The extent to which such services are available varies widely across health systems, with the VA providing relatively strong referral and linkage. Generalization to other health systems should take into account limitations of referral and availability of treatment services.

False negative screening (i.e., failure to detect a true underlying CASM condition with its respective screening tool), or unsuccessful treatment after a positive screen and diagnostic assessment, are assumed to have no effect on CASM status or other preventive healthcare. False positive screening (i.e., scoring above a threshold despite no underlying CASM condition) is assumed to lead to a negative diagnostic assessment, and therefore has no health impact in the model.

We assumed substantial rates of “spillover” benefits of CASM treatment (Supplementary Table S4) based on analysis of the Veterans Aging Cohort Study (VACS) cohort. This large longitudinal cohort follows in-care US veterans in Atlanta, Baltimore, Dallas, Houston, Los Angeles, New York, Pittsburgh, and Washington, D.C. It is predominantly male (91.2%), with people living with HIV (PLHIV) comprising half of enrolled participants. The VACS study used standardized instruments to collect quality of life, symptom, adherence, alcohol use, drug use, and health habit data, alongside medical records (pharmacy refills, laboratory, pathology, and radiology data), administrative data (service utilization, diagnostic codes), and banked biospecimens (serum, plasma, blood pellet samples). “Spillover” assumptions were derived through emulation of randomized controlled trials within VACS, with longitudinal CASM screen results as trial outcomes.

Because of the relatively limited evidence quantifying these “spillover” assumptions, results regarding life expectancy and QALY gains were analyzed in the absence of “spillover” benefits and presented as a stacked bar graph segmenting (1) the isolated health effect of improving the specific CASM condition being screened and treated, (2) “spillover” benefits to other conditions, and (3) effects of CASM improvements on broader adherence to preventive care recommendations. The first segment provides a sensitivity analysis assuming no “spillover” benefits.


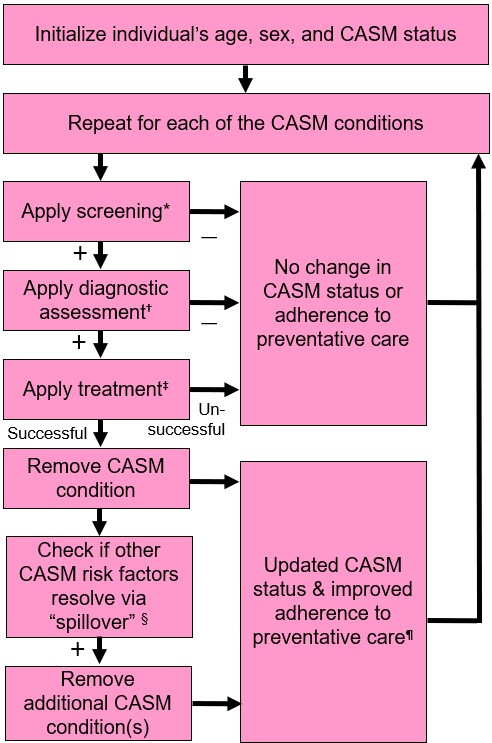


**Supplementary Figure S1: Illustration of the modeling process for applying CASM screening and treatment, and their effects on other CASM conditions and adherence to preventative care.** * Sensitivity and specificity of screening for each CASM condition is listed in Supplementary Table S2. † Sensitivity and specificity of diagnostic assessment is assumed to be 100%. ‡ Effectiveness of treatment for each CASM condition is listed in Table 1 and Supplementary Table S2. § Probability of “spillover” from each CASM condition to each other CASM condition is listed in Supplementary Table S1. ¶ Odds ratio (OR) for preventative care adherence after treatment of each CASM condition is listed in Supplementary Table S2.

**Supplementary Table S4: Estimates of probability that successful treatment of one CASM condition (rows) induces remission of other CASM conditions (columns) obtained from previous analysis of the VACS cohort using causal inference methodologies.**

| **Condition successfully treated** | **Probability of improvement of other condition** | | | | | | |
| --- | --- | --- | --- | --- | --- | --- | --- |
|  | *Depression* | *Anxiety* | *Pain* | *Alcohol* | *Tobacco* | *Opioid* | *Stimulant* |
| *Depression* | - | 0.607 (0.514–0.691) | 0.522 (0.445–0.598) | 0.186 (0.121–0.275) | 0.358 (0.261–0.466) | 0.19 (0.119–0.289) | 0.133 (0.086–0.197) |
| *Anxiety* | 0.192 (0.136–0.265) | - | 0.47 (0.386–0.556) | 0.169 (0.106–0.256) | 0.342 (0.256–0.442) | 0.266 (0.167–0.396) | 0.174 (0.11–0.267) |
| *Pain* | 0.183 (0.137–0.241) | 0.462 (0.381–0.545) | - | 0.112 (0.067–0.176) | 0.338 (0.261–0.425) | 0.339 (0.236–0.454) | 0.128 (0.084–0.191) |
| *Alcohol* | 0.284 (0.187–0.408) | 0.51 (0.364–0.604) | 0.339 (0.24–0.457) | - | 0.537 (0.41–0.662) | 0.164 (0.164–0.164) | 0.174 (0.105–0.272) |
| *Tobacco* | 0.264 (0.168–0.388) | 0.476 (0.362–0.593) | 0.343 (0.249–0.448) | 0.107 (0.055–0.199) | - | 0.183 (0.106–0.297) | 0.096 (0.054–0.167) |
| *Opioid* | 0.277 (0.189–0.389) | 0.265 (0.187–0.363) | 0.309 (0.232–0.397) | 0.124 (0.067–0.223) | 0.331 (0.233–0.45) | - | 0.130 (0.081–0.201) |
| *Stimulant* | 0.107 (0.107–0.107) | 0.399 (0.278–0.535) | 0.201 (0.128–0.300) | 0.245 (0.154–0.276) | 0.249 (0.161–0.365) | 0.246 (0.148–0.381) | - |

**Supplementary Table S5: Model assumptions and data sources.**

| **Parameter** | **Value** | **95% CI** | **Reference**^¶^ |
| --- | --- | --- | --- |
| *CASM screen sensitivity** | | | |
| Sensitivity of AUDIT alcohol screener | 0.85 | 0.73-0.91 | S1 |
| Sensitivity of PHQ-9 depression screener | 0.88 | 0.68-0.95 | S2 |
| Sensitivity of GAD-7 anxiety screener | 0.83 | 0.71-0.91 | S3 |
| Sensitivity of BPI pain screener | 0.79 | 0.71-0.86 | S4 |
| Sensitivity of FTND tobacco screener | 0.75 | 0.05-1.00 | S5 |
| Sensitivity of ASSIST opioid screener | 0.94 |  | S6 |
| Sensitivity of ASSIST stimulant screener | 0.92 |  | S6 |
| *CASM screen specificity* | | | |
| Specificity of AUDIT alcohol screener | 0.77 | 0.73-0.81 | S1 |
| Specificity of PHQ-9 depression screener | 0.88 | 0.84-0.95 | S2 |
| Specificity of GAD-7 anxiety screener | 0.84 | 0.70-0.92 | S3 |
| Specificity of BPI pain screener | 0.47 | 0.37-0.57 | S4 |
| Specificity of FTND tobacco screener | 0.80 | 0.00-0.94 | S5 |
| Specificity of ASSIST opioid screener | 0.97 |  | S6 |
| Specificity of ASSIST stimulant screener | 0.94 |  | S6 |
| *CASM condition effect on quality of life (disutility)^#^* | | | |
| Disutility of depression | 0.48 |  | S7 |
| Disutility of anxiety | 0.12 |  | S8 |
| Disutility of chronic pain | 0.41 |  | S9 |
| Disutility of unhealthy alcohol use | 0.24 |  | S10 |
| Disutility of tobacco use | 0.05 |  | S11 |
| Disutility of opioid use | 0.09 |  | S12 |
| Disutility of stimulant use | 0.06 |  | S12 |
| *CASM treatment effectiveness*^†^ |  |  |  |
| Probability alcohol misuse improves with treatment | 0.14 | 0.07**–**0.19 | S13 |
| Probability depression improves with treatment | 0.13 | 0.05**–**0.16 | S14 |
| Probability anxiety improves with treatment | 0.38 | 0.11–0.41 | S15 |
| Probability pain improves with treatment | 0.15 | 0.15–0.24 | S16 |
| Probability tobacco use improves with treatment | 0.06 | 0.02–0.08 | S17 |
| Probability opioid misuse improves with treatment | 0.24 | 0.05–0.35 | S18 |
| Probability stimulant misuse improves with treatment | 0.11 | 0.05–0.35 | S19 |
| *Probability of adherence to preventative care in absence of CASM conditions* | | | |
| Pharmacotherapy (e.g., antihypertensives)^‡^ | 0.62 |  | S20–S22 |
| Lifestyle change (e.g., weight loss)^§^ | 0.38 |  | S23–S25 |
| *Odds ratio (OR) for non-adherence to preventative care in presence of CASM conditions* | | | |
| Depression | 2.21 | 1.78–2.89 | S26–S39 |
| Anxiety | 2.04 | 1.50–2.75 | S40–S52 |
| Chronic pain | 1.34 | 1.16–1.55 | S53–S57 |
| Alcohol use disorder | 1.41 | 1.22–1.61 | S58–S72 |
| Tobacco use disorder | 1.18 | 1.03–1.37 | S73–S86 |
| Opioid use disorder | 1.18 | 0.97–1.44 | S87–S97 |
| Stimulant use disorder | 1.81 | 1.49–2.20 | S98–S105 |

*^*^* Unabbreviated names of screening tools are: Alcohol Use Disorders Identification Test (AUDIT), Patient Health Questionnaire (PHQ), Generalized Anxiety Disorder Scale (GAD), Brief Pain Inventory (BPI), Fagerström Test for Nicotine Dependence (FTND), and Alcohol, Smoking and Substance Involvement Screening Test (ASSIST).

*^#^* Disutility denotes the impact of having a CASM on quality of life on a 0 to 1 scale, with 0 being no impact and 1 being impact so severe it is equivalent to death.

^†^ A 35% diminution of effectiveness is applied to efficacy estimates from randomized controlled trials (RCTs) in order to capture more modest effectiveness in clinical practice compared to RCTs. A 35% diminution of effectiveness is applied to efficacy estimates from randomized controlled trials (RCTs) in order to capture more modest effectiveness in clinical practice compared to RCTs. The 35% is a midpoint of the range of diminution levels reported in the literature we reviewed, with standardized effect sizes of psychological therapies attenuating between 24% to 29% in clinical practice compared to RCTs (Reference S81), and the effect sizes of dietary therapies attenuating by a median of 41% in clinical practice compared to RCTs (Reference S82).

‡Applies to cardiovascular disease, diabetes, hepatitis C, HIV, hypertension, hyperlipidemia, and preventative immunizations.

^§^Applies to inadequate exercise, unhealthy diet, obesity (BMI>30), and bipolar disorder.

^¶^References with a prefix “S” are supplied in the Supplementary References appendix.

**Supplementary Results**


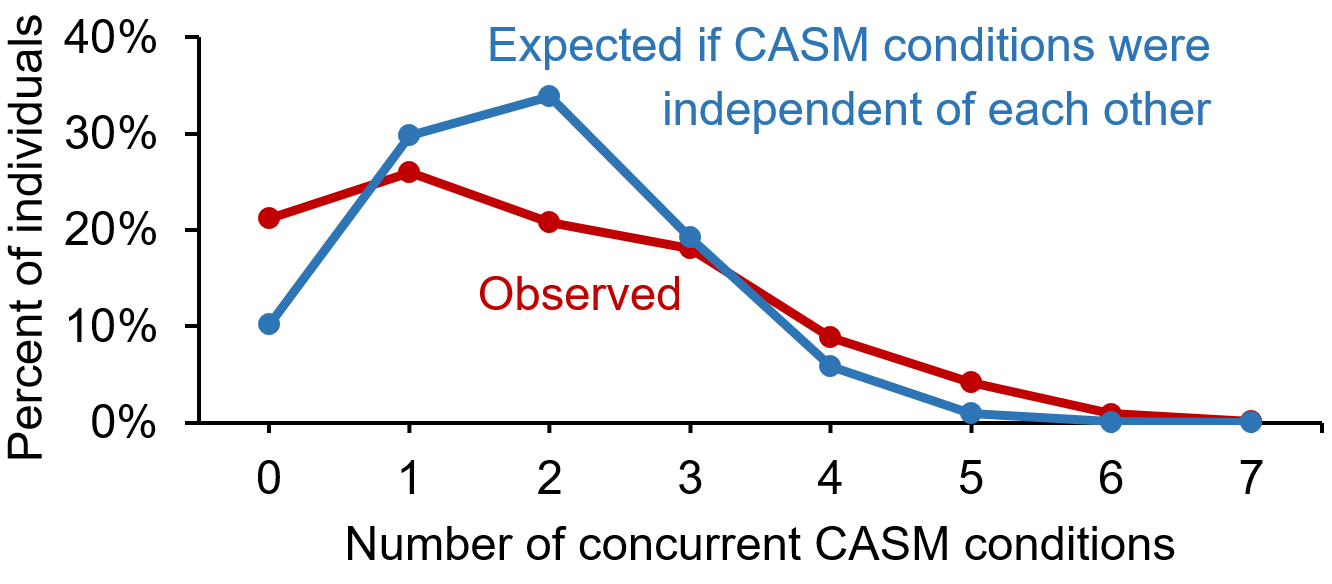


**Supplementary Figure S2: Comparison of observed CASM co-occurrence to expected CASM co-occurrence if conditions were independent of each other.** Expected co-occurrence of independent conditions is calculated by multiplying the prevalence of having each condition, P_condition_, or not having each condition (1- P_condition_), as independent probabilities. For example, the expected number of individuals with zero CASM conditions, if the conditions were independent of each other ,is calculated as (1-P_depression_)*(1-P_anxiety_)*(1-P_pain_)*(1-P_alcohol_)*(1-P_tobacco_)*(1-P_opioids_)*(1-P_stimulants_). We sum the probabilities of all seven ways an individual can have one or six conditions, 21 ways an individual can have two or five conditions, and 35 ways an individual can have three or four conditions in order to obtain the total expected percent of individuals having the indicated number of conditions.


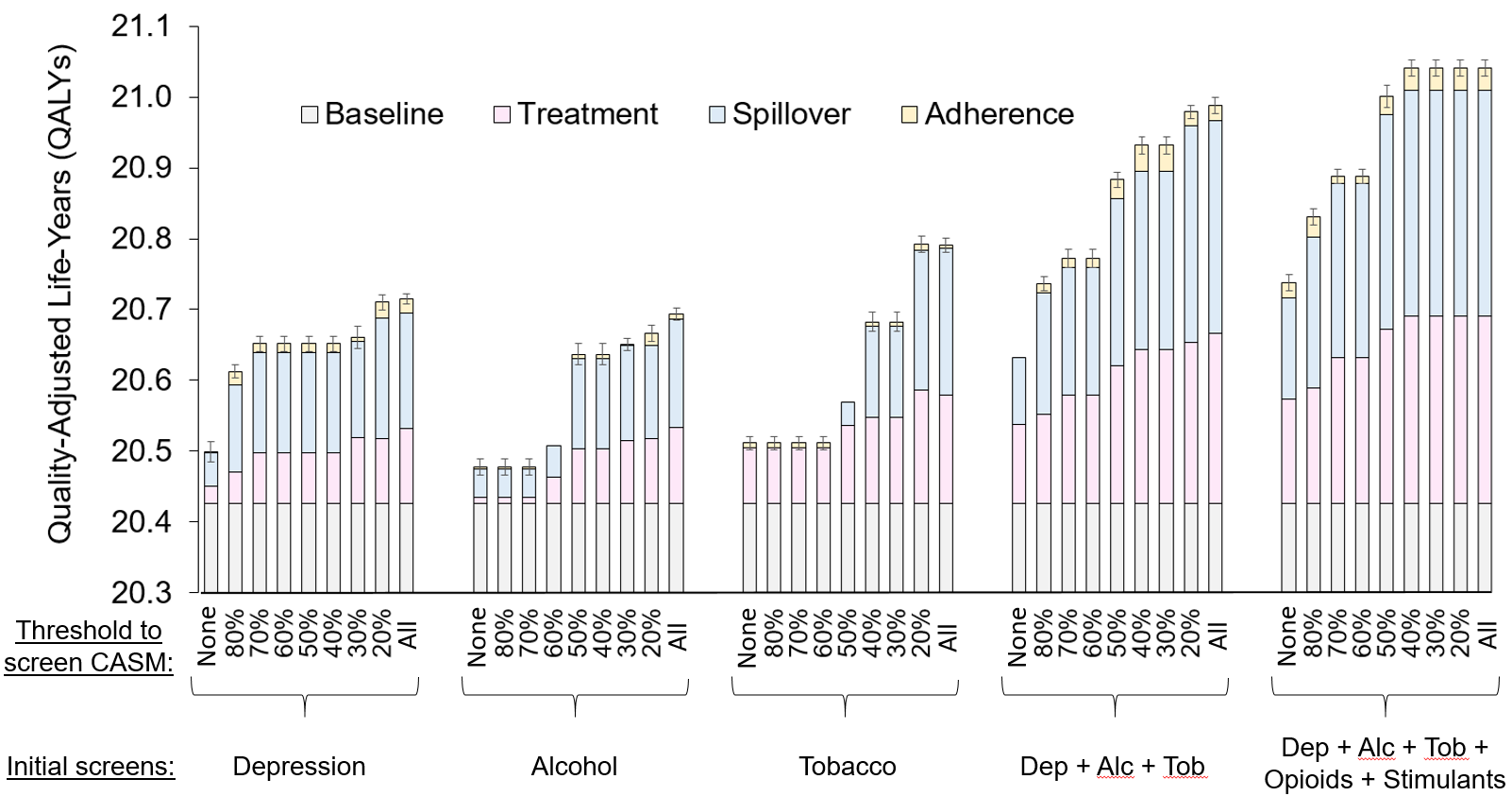


**Supplementary Figure S3: Impact on quality-adjusted life-years (QALYs) of screening co-occurring alcohol, substance, and mood-related conditions (CASM)**. All strategies begin with screening an initial set of conditions recommended for US primary care, shown at the bottom of the figure. “None” denotes screening only these individual conditions without further screening of conditions that are likely to co-occur. “All” denotes screening all seven CASM conditions if any of the initially screened conditions screen positive. Percentages indicate the minimum probability of co-occurrence, above which another condition is screened if the initially screened condition screens positive. For example, at a threshold of 80%, only conditions with ≥80% probability of co-occurring with a positive condition are further screened, e.g., anxiety is screened if depression screens positive. QALY gains are subdivided by mechanism into: (pink) direct effect of treating the screened conditions, (blue) “spillover” effect onto other CASM conditions due to causal associations in their remission, and (yellow) improved adherence to chronic care such as maintenance medications.


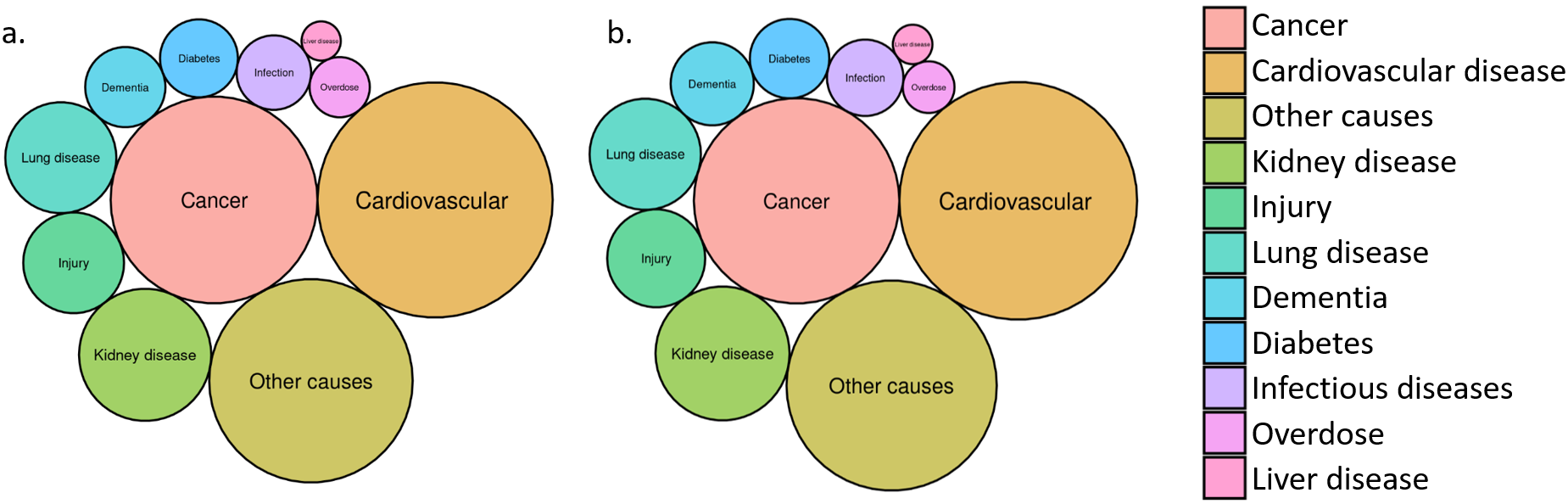


**Supplementary Figure S4:** Bubble cloud showing major causes of death in the baseline cohort (left) and the cohort after the maximal strategy of screening all seven CASM conditions (depression, anxiety, chronic pain, and unhealthy alcohol, tobacco, opioid, and stimulant use). Bubble areas denote the proportion of deaths from each category of causes. For ease of visualization, categories have been simplified as follows from the full model: overdose combines overdoses from oral opioids, oral stimulants, and intravenous drugs. Injury combines accidental injury, homicide, and suicide. Cardiovascular combines heart diseases and stroke. Infectious diseases combines HIV, hepatitis, and respiratory illnesses. Dementia combines Alzheimer’s and Parkinson’s.


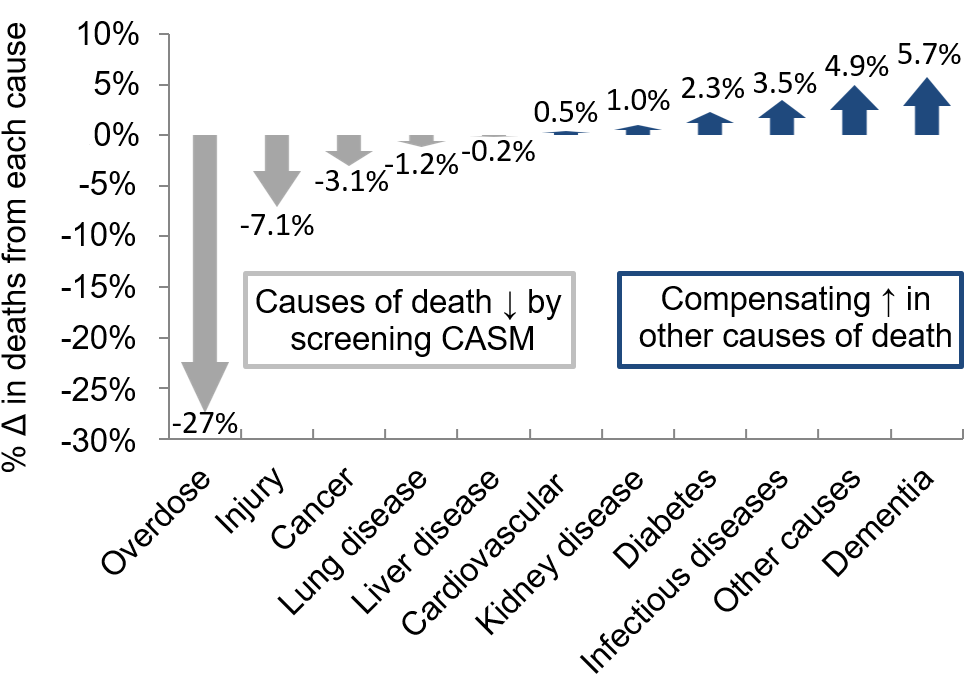


**Supplementary Figure S5: Effect of maximal CASM screening on the distribution of causes of death.** Arrows show the percent change in the number of deaths observed for each cause, comparing a counterfactual of no CASM screening to maximal strategy of screening all seven CASM conditions (depression, anxiety, chronic pain, and unhealthy alcohol, tobacco, opioid, and stimulant use). Categories have been simplified as follows: overdose combines overdoses from oral opioids, oral stimulants, and intravenous drugs. Injury combines accidental injury, homicide, and suicide. Cardiovascular combines heart diseases and stroke. Infectious diseases combines HIV, hepatitis, and respiratory illnesses. Dementia combines Alzheimer’s and Parkinson’s.


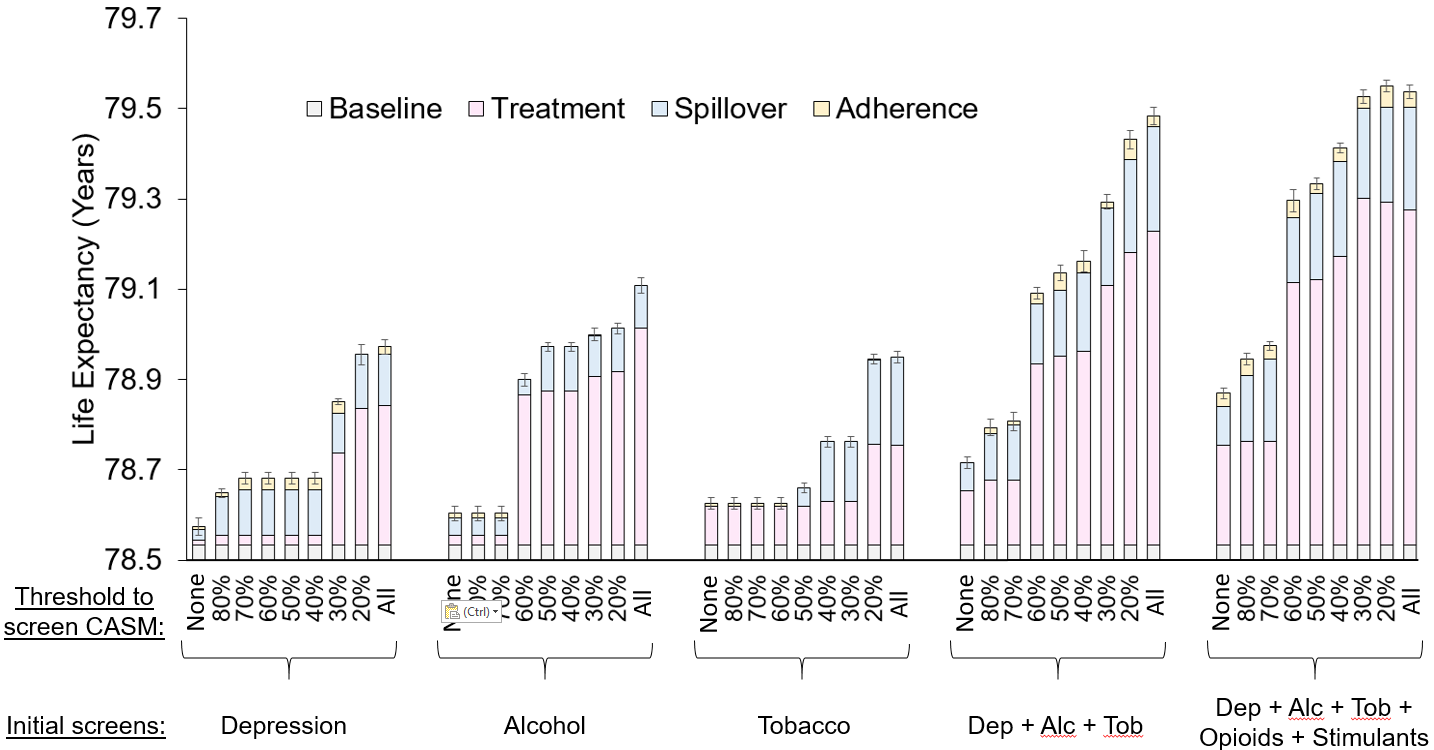


**Supplementary Figure S6: Impact on life expectancy of diagnostic assessment of co-occurring alcohol, substance, and mood-related conditions (CASM).** All strategies begin with screening an initial set of conditions recommended for US primary care, shown at the bottom of the figure. “None” denotes screening only these conditions, with diagnostic assessment of those screening positive, but no further assessment of conditions that are likely to co-occur. “All” denotes conducting a gold-standard diagnostic assessment of all seven CASM conditions if any of the initially screened conditions screen positive. Percentages indicate the minimum probability of co-occurrence, above which another condition receives diagnostic assessment if an initially screened condition screens positive. For example, at a threshold of 80%, only conditions with ≥80% probability of co-occurring with a positive condition receive diagnostic assessment, e.g., diagnostic assessment for anxiety is conducted if depression screens positive. Life expectancy gains are subdivided by mechanism into: (pink) direct effect of treating the screened conditions, (blue) “spillover” effect onto other CASM conditions due to causal associations in their remission, and (yellow) improved adherence to chronic care such as maintenance medications.

**Supplementary Discussion**

Implications of the study:

Here we expand on additional implications of the study, including constraints of current health systems and practices for CASM screening and referral/treatment, and broader healthcare reforms needed to address CASM.

Our findings have the potential to inform efforts to increase CASM screening in the US. Screening for a subset CASM conditions – most commonly unhealthy alcohol use, tobacco use, and/or depression – is already mandated in some larger US health systems. However, constraints of current systems and practices, including very limited provider time for preventive care encounters, make it challenging for systems to consistently screen all clients for seven or more CASM conditions. Ultimately, maximizing the impact of CASM screening will likely require systemic healthcare reforms that incentivize comprehensive CASM screening and care due to the value it provides to clients and society. Our study examined a more limited reform, whereby widely-implemented screens are followed by less-often-implemented screens when the probability of co-occurring conditions in the client population exceeds a threshold. The threshold could be selected, in part, based on local health system constraints. While potentially more immediately actionable, this approach should be viewed as only a stopgap for systemic preventive care strengthening.

Limitations of the study:

This study has several important limitations.

First, our study cohort consisted of US military veterans who were predominantly male and over age 30. Results of this study may not be generalizable to other populations, especially women and youth. Subgroup analyses involving these populations would have strengthened the study, but will require longitudinal CASM measurements in cohorts in which these subgroups are better-represented. By using probability-based thresholds for CASM screening rather than specifying conditions for follow-on assessment, the proposed approach can be customized to populations with similar CASM effect sizes, but such extensions should be performed with extreme caution if they involve groups under-represented in our analysis.

Second, our study did not incorporate time and resource constraints. These include the possibility that CASM screening might displace other forms of healthcare due to limited provider time, specialists to perform diagnosis and treatment, or financial constraints on the part of healthcare payers or out-of-pocket cost burden to patients.

Third, our study was limited in scope. We focused on only seven highly-prevalent CASM conditions. Each condition was treated monolithically, e.g., cocaine use disorders were bundled together with a monolithic representation of stimulant use disorder, and the multitude of disorders related to prescription drug misuse were only represented insofar as they overlapped with CASM (e.g., prescription opioids as part of opioid use disorder). We did not model cannabis use disorders, which are prevalent but suffer from a dearth of high-quality longitudinal evidence. Our model did not include post-traumatic stress disorder (PTSD), a condition that is extremely common in military veterans and should be routinely screened and treated. Future work should investigate how PTSD diagnosis and treatment outcomes could guide CASM screening.

Fourth, we modeled strategies in which all patients screening positive are referred to evidence-based treatment options, which may be challenging to achieve in practice. We did not systematically vary other important components of the CASM care continuum such as the choice of screening instruments, referral mechanisms, diagnostic assessment or treatment methods, or strategies to support treatment adherence, retention, or re-engagement. The limited scope of this study is not meant to imply that screening decisions are the sole or most important factor in strengthening CASM treatment.

Fifth, we relied on relatively limited evidence quantifying “spillover” benefits from successful treatment of one CASM condition to relief from other conditions. Because reliance on limited evidence could bias results, we (1) conducted sensitivity analyses across a wide range of “spillover” effect sizes, and (2) reported results for all screening scenarios under the more conservative assumption of no existence of “spillover.”

Finally, like all modeling studies, we made simplifying assumptions, including neglecting the potential correlation in the sensitivity of different screens in the same patient; neglecting the possibility of misdiagnosis by treating diagnostic assessment as a gold standard; and neglecting the time elapsed between screening, diagnosis, treatment, and remission – which were assumed to be short relative to LE. However, we conducted extensive sensitivity analyses to multiple sources of uncertainty. Results warrant confirmation in the context of trials and implementation studies, and should be evaluated for feasibility and acceptability in real-world settings.

**Supplementary References**

1. McNeely J, Strauss SM, Saitz R, Cleland CM, Palamar JJ, Rotrosen J, et al. A Brief Patient Self-administered Substance Use Screening Tool for Primary Care: Two-site Validation Study of the Substance Use Brief Screen (SUBS). Am J Med. 2015 July;128(7):784.e9-784.e19.
2. Kroenke K, Spitzer RL, Williams JBW. The PHQ-9: Validity of a brief depression severity measure. J Gen Intern Med. 2001 Sept;16(9):606–13.
3. Plummer F, Manea L, Trepel D, McMillan D. Screening for anxiety disorders with the GAD-7 and GAD-2: a systematic review and diagnostic metaanalysis. Gen Hosp Psychiatry. 2016 Mar;39:24–31.
4. Erdemoglu ÂK, Koc R. Brief Pain Inventory score identifying and discriminating neuropathic and nociceptive pain. Acta Neurol Scand. 2013 Apr;n/a-n/a.
5. Mikami I, Akechi T, Kugaya A, Okuyama T, Nakano T, Okamura H, et al. Screening for Nicotine Dependence among Smoking-related Cancer Patients. Jpn J Cancer Res. 1999 Oct;90(10):1071–5.
6. Humeniuk R, Ali R, World Health Organization. ASSIST Phase II Study Group. Validation of the Alcohol, Smoking and Substance Involvement Screening Test (ASSIST) and pilot brief intervention [electronic resource] : a technical report of phase II findings of the WHO ASSIST Project / prepared by Rachel Humeniuk & Robert Ali, on behalf of the WHO ASSIST Phase II Study Group. 2006; Available from: https://apps.who.int/iris/handle/10665/43504
7. Mohiuddin S, Payne K. Utility Values for Adults with Unipolar Depression: Systematic Review and Meta-Analysis. Med Decis Mak Int J Soc Med Decis Mak. 2014 July;34(5):666–85.
8. Stein MB, Roy-Byrne PP, Craske MG, Bystritsky A, Sullivan G, Pyne JM, et al. Functional impact and health utility of anxiety disorders in primary care outpatients. Med Care. 2005 Dec;43(12):1164–70.
9. Wetherington S, Delong L, Kini S, Veledar E, Schaufele MK, McKenzie-Brown AM, et al. Pain quality of life as measured by utilities. Pain Med Malden Mass. 2014 May;15(5):865–70.
10. Kraemer KL, Roberts MS, Horton NJ, Palfai T, Samet JH, Freedner N, et al. Health utility ratings for a spectrum of alcohol-related health states. Med Care. 2005 June;43(6):541–50.
11. Vogl M, Wenig CM, Leidl R, Pokhrel S. Smoking and health-related quality of life in English general population: implications for economic evaluations. BMC Public Health. 2012 Mar 19;12:203.
12. Aden B, Dunning A, Nosyk B, Wittenberg E, Bray JW, Schackman BR. Impact of Illicit Drug Use on Health-Related Quality of Life in Opioid-Dependent Patients Undergoing HIV Treatment. J Acquir Immune Defic Syndr 1999. 2015 Nov 1;70(3):304–10.
13. Fleming MF, Barry KL, Manwell LB, Johnson K, London R. Brief physician advice for problem alcohol drinkers. A randomized controlled trial in community-based primary care practices. JAMA. 1997 Apr 2;277(13):1039–45.
14. Dietrich AJ, Oxman TE, Williams JW, Schulberg HC, Bruce ML, Lee PW, et al. Re-engineering systems for the treatment of depression in primary care: cluster randomised controlled trial. BMJ. 2004 Sept 11;329(7466):602.
15. Villabø MA, Narayanan M, Compton SN, Kendall PC, Neumer SP. Cognitive–behavioral therapy for youth anxiety: An effectiveness evaluation in community practice. J Consult Clin Psychol. 2018 Sept;86(9):751–64.
16. Skillgate E, Pico-Espinosa OJ, Côté P, Jensen I, Viklund P, Bottai M, et al. Effectiveness of deep tissue massage therapy, and supervised strengthening and stretching exercises for subacute or persistent disabling neck pain. The Stockholm Neck (STONE) randomized controlled trial. Musculoskelet Sci Pract. 2020 Feb;45:102070.
17. Hartmann-Boyce J, Chepkin SC, Ye W, Bullen C, Lancaster T. Nicotine replacement therapy versus control for smoking cessation. Cochrane Tobacco Addiction Group, editor. Cochrane Database Syst Rev [Internet]. 2018 May 31 [cited 2021 Nov 15];2019(1). Available from: http://doi.wiley.com/10.1002/14651858.CD000146.pub5
18. Schwartz RP, Highfield DA, Jaffe JH, Brady JV, Butler CB, Rouse CO, et al. A Randomized Controlled Trial of Interim Methadone Maintenance. Arch Gen Psychiatry. 2006 Jan 1;63(1):102.
19. Baker A, Lee NK, Claire M, Lewin TJ, Grant T, Pohlman S, et al. Brief cognitive behavioural interventions for regular amphetamine users: a step in the right direction. Addiction. 2005 Mar;100(3):367–78.
20. Durand H, Hayes P, Morrissey EC, Newell J, Casey M, Murphy AW, et al. Medication adherence among patients with apparent treatment-resistant hypertension: systematic review and meta-analysis. J Hypertens. 2017 Dec;35(12):2346–57.
21. Sartori AC, Rodrigues Lucena TF, Lopes CT, Picinin Bernuci M, Yamaguchi MU. Educational Intervention Using WhatsApp on Medication Adherence in Hypertension and Diabetes Patients: A Randomized Clinical Trial. Telemed J E-Health Off J Am Telemed Assoc. 2020 Dec;26(12):1526–32.
22. Hedegaard U, Kjeldsen LJ, Pottegård A, Henriksen JE, Lambrechtsen J, Hangaard J, et al. Improving Medication Adherence in Patients with Hypertension: A Randomized Trial. Am J Med. 2015 Dec;128(12):1351–61.
23. Friedrich M, Gittler G, Halberstadt Y, Cermak T, Heiller I. Combined exercise and motivation program: effect on the compliance and level of disability of patients with chronic low back pain: a randomized controlled trial. Arch Phys Med Rehabil. 1998 May;79(5):475–87.
24. Reilly K, Lovejoy B, Williams R, Roth H. Differences between a supervised and independent strength and conditioning program with chronic low back syndromes. J Occup Med Off Publ Ind Med Assoc. 1989 June;31(6):547–50.
25. Härkäpää K, Järvikoski A, Mellin G, Hurri H, Luoma J. Health locus of control beliefs and psychological distress as predictors for treatment outcome in low-back pain patients: results of a 3-month follow-up of a controlled intervention study. Pain. 1991 July;46(1):35–41.
26. Adeoti AO, Dada M, Elebiyo T, Fadare J, Ojo O. Survey of antiretroviral therapy adherence and predictors of poor adherence among HIV patients in a tertiary institution in Nigeria. Pan Afr Med J. 2019 Jul 31;33:277. doi: 10.11604/pamj.2019.33.277.18711. PMID: 31692880; PMCID: PMC6815489.
27. Onono M, Odwar T, Abuogi L, Owuor K, Helova A, Bukusi E, Turan J, Hampanda K. Effects of Depression, Stigma and Intimate Partner Violence on Postpartum Women's Adherence and Engagement in HIV Care in Kenya. AIDS Behav. 2020 Jun;24(6):1807-1815. doi: 10.1007/s10461-019-02750-y. PMID: 31813076; PMCID: PMC7228848.
28. Camargo CC, Cavassan NRV, Tasca KI, Meneguin S, Miot HA, Souza LR. Depression and Coping Are Associated with Failure of Adherence to Antiretroviral Therapy Among People Living with HIV/AIDS. AIDS Res Hum Retroviruses. 2019 Nov/Dec;35(11-12):1181-1188. doi: 10.1089/aid.2019.0050. Epub 2019 Oct 8. PMID: 31592722.
29. Yu, Y., Luo, D., Chen, X. et al. Medication adherence to antiretroviral therapy among newly treated people living with HIV. BMC Public Health 18, 825 (2018). https://doi.org/10.1186/s12889-018-5731-z
30. Kinyanda E, Nakasujja N, Levin J, Birabwa H, Mpango R, Grosskurth H, Seedat S, Patel V. Major depressive disorder and suicidality in early HIV infection and its association with risk factors and negative outcomes as seen in semi-urban and rural Uganda. J Affect Disord. 2017 Apr 1;212:117-127. doi: 10.1016/j.jad.2017.01.033. Epub 2017 Jan 23. PMID: 28160684.
31. Moraes RP, Casseb J. Depression and adherence to antiretroviral treatment in HIV-positive men in São Paulo, the largest city in South America: Social and psychological implications. Clinics (Sao Paulo). 2017 Dec;72(12):743-749. doi: 10.6061/clinics/2017(12)05. PMID: 29319720; PMCID: PMC5738567.
32. Gebrezgabher, B.B., Kebede, Y., Kindie, M. et al. Determinants to antiretroviral treatment non-adherence among adult HIV/AIDS patients in northern Ethiopia. AIDS Res Ther 14, 16 (2017). https://doi.org/10.1186/s12981-017-0143-1
33. Mohammed H, Kieltyka L, Richardson-Alston G, Magnus M, Fawal H, Vermund SH, Rice J, Kissinger P. Adherence to HAART among HIV-infected persons in rural Louisiana. AIDS Patient Care STDS. 2004 May;18(5):289-96. doi: 10.1089/108729104323076025. PMID: 15186712.
34. Tao J, Qian HZ, Kipp AM, Ruan Y, Shepherd BE, Amico KR, Shao Y, Lu H, Vermund SH. Effects of depression and anxiety on antiretroviral therapy adherence among newly diagnosed HIV-infected Chinese MSM. AIDS. 2017 Jan 28;31(3):401-406. doi: 10.1097/QAD.0000000000001287. PMID: 27677168; PMCID: PMC5233466.
35. Letta S, Demissie A, Oljira L, Dessie Y. Factors associated with adherence to Antiretroviral Therapy (ART) among adult people living with HIV and attending their clinical care, Eastern Ethiopia. BMC Int Health Hum Rights. 2015 Dec 28;15:33. doi: 10.1186/s12914-015-0071-x. Erratum in: BMC Int Health Hum Rights. 2016;16:8. PMID: 26711659; PMCID: PMC4693416.
36. Memiah P, Shumba C, Etienne-Mesubi M, Agbor S, Hossain MB, Komba P, Niyang M, Biadgilign S. The effect of depressive symptoms and CD4 count on adherence to highly active antiretroviral therapy in sub-Saharan Africa. J Int Assoc Provid AIDS Care. 2014 Jul-Aug;13(4):346-52. doi: 10.1177/2325957413503368. PMID: 24114726.
37. Do, H.M., Dunne, M.P., Kato, M. et al. Factors associated with suboptimal adherence to antiretroviral therapy in Viet Nam: a cross-sectional study using audio computer-assisted self-interview (ACASI). BMC Infect Dis 13, 154 (2013). https://doi.org/10.1186/1471-2334-13-154
38. Li L, Lee SJ, Wen Y, Lin C, Wan D, Jiraphongsa C. Antiretroviral therapy adherence among patients living with HIV/AIDS in Thailand. Nurs Health Sci. 2010 Jun;12(2):212-20. doi: 10.1111/j.1442-2018.2010.00521.x. PMID: 20602694; PMCID: PMC2947817.
39. Royal SW, Kidder DP, Patrabansh S, Wolitski RJ, Holtgrave DR, Aidala A, Pals S, Stall R. Factors associated with adherence to highly active antiretroviral therapy in homeless or unstably housed adults living with HIV. AIDS Care. 2009 Apr;21(4):448-55. doi: 10.1080/09540120802270250. PMID: 19401865.
40. Adeoti AO, Dada M, Elebiyo T, Fadare J, Ojo O. Survey of antiretroviral therapy adherence and predictors of poor adherence among HIV patients in a tertiary institution in Nigeria. Pan Afr Med J. 2019 Jul 31;33:277. doi: 10.11604/pamj.2019.33.277.18711. PMID: 31692880; PMCID: PMC6815489.
41. Been SK, Schadé A, Bassant N, Kastelijns M, Pogány K, Verbon A. Anxiety, depression and treatment adherence among HIV-infected migrants. AIDS Care. 2019 Aug;31(8):979-987. doi: 10.1080/09540121.2019.1601676. Epub 2019 Apr 8. PMID: 30957530.
42. Pokhrel KN, Pokhrel KG, Sharma VD, Poudel KC, Neupane SR, Mlunde LB, Jimba M. Mental health disorders and substance use among people living with HIV in Nepal: their influence on non-adherence to anti-retroviral therapy. AIDS Care. 2019 Aug;31(8):923-931. doi: 10.1080/09540121.2019.1587365. Epub 2019 Mar 5. PMID: 30835503.
43. Nguyen NT, Tran BX, Hwang LY, Markham CM, Swartz MD, Vidrine JI, Phan HT, Latkin CA, Vidrine DJ. Effects of cigarette smoking and nicotine dependence on adherence to antiretroviral therapy among HIV-positive patients in Vietnam. AIDS Care. 2016;28(3):359-64. doi: 10.1080/09540121.2015.1090535. Epub 2015 Oct 13. PMID: 26461976; PMCID: PMC6691494.
44. Joshi B, Chauhan S, Pasi A, Kulkarni R, Sunil N, Bachani D, Mankeshwar R; ART adherence study group. Level of suboptimal adherence to first line antiretroviral treatment & its determinants among HIV positive people in India. Indian J Med Res. 2014 Jul;140(1):84-95. PMID: 25222782; PMCID: PMC4181166.
45. Nilsson Schönnesson L, Williams ML, Ross MW, Bratt G, Keel B. Factors associated with suboptimal antiretroviral therapy adherence to dose, schedule, and dietary instructions. AIDS Behav. 2007 Mar;11(2):175-83. doi: 10.1007/s10461-006-9160-0. PMID: 16927178.
46. Ingersoll K. The impact of psychiatric symptoms, drug use, and medication regimen on non-adherence to HIV treatment. AIDS Care. 2004 Feb;16(2):199-211. doi: 10.1080/09540120410001641048. PMID: 14676026.
47. Guimarães MD, Rocha GM, Campos LN, de Freitas FM, Gualberto FA, Teixeira Rd, de Castilho FM. Difficulties reported by HIV-infected patients using antiretroviral therapy in Brazil. Clinics (Sao Paulo). 2008 Apr;63(2):165-72. doi: 10.1590/s1807-59322008000200003. PMID: 18438569; PMCID: PMC2664217.
48. Nel A, Kagee A. The relationship between depression, anxiety and medication adherence among patients receiving antiretroviral treatment in South Africa. AIDS Care. 2013 Aug;25(8):948-55. doi: 10.1080/09540121.2012.748867. Epub 2012 Dec 11. PMID: 23231527.
49. Tucker JS, Burnam MA, Sherbourne CD, Kung FY, Gifford AL. Substance use and mental health correlates of nonadherence to antiretroviral medications in a sample of patients with human immunodeficiency virus infection. Am J Med. 2003 May;114(7):573-80. doi: 10.1016/s0002-9343(03)00093-7. PMID: 12753881.
50. Adejumo O, Oladeji B, Akpa O, et al. Psychiatric disorders and adherence to antiretroviral therapy among a population of HIV-infected adults in Nigeria. Int J STD AIDS. 2016;27(11):938-949. doi:10.1177/0956462415600582
51. Prasithsirikul W, Chongthawonsatid S, Ohata PJ, Keadpudsa S, Klinbuayaem V, Rerksirikul P, Kerr SJ, Ruxrungtham K, Ananworanich J, Avihingsanon A; PROGRESS study team. Depression and anxiety were low amongst virally suppressed, long-term treated HIV-infected individuals enrolled in a public sector antiretroviral program in Thailand. AIDS Care. 2017 Mar;29(3):299-305. doi: 10.1080/09540121.2016.1201194. Epub 2016 Aug 10. PMID: 27683949.
52. Yu Y, Luo D, Chen X, Huang Z, Wang M, Xiao S. Medication adherence to antiretroviral therapy among newly treated people living with HIV. BMC Public Health. 2018;18(1):825. Published 2018 Jul 4. doi:10.1186/s12889-018-5731-z
53. Denis, C. M., Morales, K. H., Wu, Q., Metzger, D. S., & Cheatle, M. D. (2019). Association Between Diagnoses of Chronic Noncancer Pain, Substance Use Disorder, and HIV-Related Outcomes in People Living With HIV. Journal of acquired immune deficiency syndromes (1999), 82 Suppl 2(2), S142–S147. https://doi.org/10.1097/QAI.0000000000002179 (study 13)
54. Nguyen, N. T., Tran, B. X., Hwang, L. Y., Markham, C. M., Swartz, M. D., Vidrine, J. I., Phan, H. T., Latkin, C. A., & Vidrine, D. J. (2016). Effects of cigarette smoking and nicotine dependence on adherence to antiretroviral therapy among HIV-positive patients in Vietnam. AIDS care, 28(3), 359–364. https://doi.org/10.1080/09540121.2015.1090535 (study 25)
55. Merlin, J. S., Westfall, A. O., Raper, J. L., Zinski, A., Norton, W. E., Willig, J. H., Gross, R., Ritchie, C. S., Saag, M. S., & Mugavero, M. J. (2012). Pain, mood, and substance abuse in HIV: implications for clinic visit utilization, antiretroviral therapy adherence, and virologic failure. Journal of acquired immune deficiency syndromes (1999), 61(2), 164–170. https://doi.org/10.1097/QAI.0b013e3182662215 (study 53)
56. Shah, B., Walshe, L., Saple, D. G., Mehta, S. H., Ramnani, J. P., Kharkar, R. D., Bollinger, R. C., & Gupta, A. (2007). Adherence to antiretroviral therapy and virologic suppression among HIV-infected persons receiving care in private clinics in Mumbai, India. Clinical infectious diseases : an official publication of the Infectious Diseases Society of America, 44(9), 1235–1244. https://doi.org/10.1086/513429 (study 57)
57. Johnson, M. O., Charlebois, E., Morin, S. F., Catz, S. L., Goldstein, R. B., Remien, R. H., Rotheram-Borus, M. J., Mickalian, J. D., Kittel, L., Samimy-Muzaffar, F., Lightfoot, M. A., Gore-Felton, C., Chesney, A., & NIMH Healthy Living Project Team (2005). Perceived adverse effects of antiretroviral therapy. Journal of pain and symptom management, 29(2), 193–205. https://doi.org/10.1016/j.jpainsymman.2004.05.005 (study 91)
58. Soares, R., Brito, A. M., Lima, K., & Lapa, T. M. (2019). Adherence to antiretroviral therapy among people living with HIV/AIDS in northeastern Brazil: a cross-sectional study. Sao Paulo medical journal = Revista paulista de medicina, 137(6), 479–485. https://doi.org/10.1590/1516-3180.2019.0212170919 (study 4)
59. King, D., Grasso, C., Dant, L., Elsesser, S. A., Crane, H. M., Cropsey, K. L., Mayer, K. H., & O'Cleirigh, C. (2018). Treatment Outcomes Associated with Quitting Cigarettes Among Sexual Minority Men Living with HIV: Antiretroviral Adherence, Engagement in Care, and Sustained HIV RNA Suppression. AIDS and behavior, 22(9), 2868–2876. https://doi.org/10.1007/s10461-018-2116-3 (study 35)
60. Nolan, S., Walley, A. Y., Heeren, T. C., Patts, G. J., Ventura, A. S., Sullivan, M. M., Samet, J. H., & Saitz, R. (2017). HIV-infected individuals who use alcohol and other drugs, and virologic suppression. AIDS care, 29(9), 1129–1136. https://doi.org/10.1080/09540121.2017.1327646 (study 49)
61. Aye, W. L., Puckpinyo, A., & Peltzer, K. (2017). Non-adherence to anti-retroviral therapy among HIV infected adults in Mon State of Myanmar. BMC public health, 17(1), 391. https://doi.org/10.1186/s12889-017-4309-5 (study 50)
62. Cioe, P. A., Gamarel, K. E., Pantalone, D. W., Monti, P. M., Mayer, K. H., & Kahler, C. W. (2017). Cigarette Smoking and Antiretroviral Therapy (ART) Adherence in a Sample of Heavy Drinking HIV-Infected Men Who Have Sex with Men (MSM). AIDS and behavior, 21(7), 1956–1963. https://doi.org/10.1007/s10461-016-1496-5 (study 66)
63. Nguyen, N. T., Tran, B. X., Hwang, L. Y., Markham, C. M., Swartz, M. D., Vidrine, J. I., Phan, H. T., Latkin, C. A., & Vidrine, D. J. (2016). Effects of cigarette smoking and nicotine dependence on adherence to antiretroviral therapy among HIV-positive patients in Vietnam. AIDS care, 28(3), 359–364. https://doi.org/10.1080/09540121.2015.1090535 (study 72)
64. Ompad, D. C., Kingdon, M., Kupprat, S., Halkitis, S. N., Storholm, E. D., & Halkitis, P. N. (2014). Smoking and HIV-related health issues among older HIV-positive gay, bisexual, and other men who have sex with men. Behavioral medicine (Washington, D.C.), 40(3), 99–107. https://doi.org/10.1080/08964289.2014.889067 (study 89)
65. Degroote, S., Vogelaers, D., Vermeir, P., Mariman, A., De Rick, A., Van Der Gucht, B., Pelgrom, J., Van Wanzeele, F., Verhofstede, C., Vancauwenberghe, J., & Vandijck, D. (2014). Determinants of adherence in a cohort of Belgian HIV patients: a pilot study. Acta clinica Belgica, 69(2), 111–115. https://doi.org/10.1179/0001551214Z.00000000035 (study 93)
66. Batista, J. d., Albuquerque, M., Santos, M. L., Miranda-Filho, D., Lacerda, H. R., Maruza, M., Moura, L. V., Coimbra, I., & Ximenes, R. A. (2014). Association between smoking, crack cocaine abuse and the discontinuation of combination antiretroviral therapy in Recife, Pernambuco, Brazil. Revista do Instituto de Medicina Tropical de Sao Paulo, 56(2), 127–132. https://doi.org/10.1590/S0036-46652014000200007 (study 94)
67. Sharma, S., Khadga, P., Dhungana, G. P., & Chitrakar, U. (2013). Medication adherence to antiretroviral therapy among patients visiting antiretroviral therapy center at Tribhuvan University Teaching Hospital, Kathmandu Nepal. Kathmandu University medical journal (KUMJ), 11(41), 50–53. https://doi.org/10.3126/kumj.v11i1.11027 (study 103)
68. Murri, R., Guaraldi, G., Lupoli, P., Crisafulli, R., Marcotullio, S., von Schloesser, F., & Wu, A. W. (2009). Rate and predictors of self-chosen drug discontinuations in highly active antiretroviral therapy-treated HIV-positive individuals. AIDS patient care and STDs, 23(1), 35–39. https://doi.org/10.1089/apc.2007.0248 (study 132)
69. Mellins, C. A., Chu, C., Malee, K., Allison, S., Smith, R., Harris, L., Higgins, A., Zorrilla, C., Landesman, S., Serchuck, L., & Larussa, P. (2008). Adherence to antiretroviral treatment among pregnant and postpartum HIV-infected women. AIDS care, 20(8), 958–968. https://doi.org/10.1080/09540120701767208 (study 134)
70. Cohn, S. E., Umbleja, T., Mrus, J., Bardeguez, A. D., Andersen, J. W., & Chesney, M. A. (2008). Prior illicit drug use and missed prenatal vitamins predict nonadherence to antiretroviral therapy in pregnancy: adherence analysis A5084. AIDS patient care and STDs, 22(1), 29–40. https://doi.org/10.1089/apc.2007.0053 (study 139)
71. Spire, B., Duran, S., Souville, M., Leport, C., Raffi, F., Moatti, J. P., & APROCO cohort study group (2002). Adherence to highly active antiretroviral therapies (HAART) in HIV-infected patients: from a predictive to a dynamic approach. Social science & medicine (1982), 54(10), 1481–1496. https://doi.org/10.1016/s0277-9536(01)00125-3 (study 156)
72. Azar, P., Wood, E., Nguyen, P., Luma, M., Montaner, J., Kerr, T., & Milloy, M. J. (2015). Drug use patterns associated with risk of non-adherence to antiretroviral therapy among HIV-positive illicit drug users in a Canadian setting: a longitudinal analysis. BMC infectious diseases, 15, 193. https://doi.org/10.1186/s12879-015-0913-0 (study 196)
73. Cohn, S. E., Jiang, H., McCutchan, J. A., Koletar, S. L., Murphy, R. L., Robertson, K. R., de St Maurice, A. M., Currier, J. S., & Williams, P. L. (2011). Association of ongoing drug and alcohol use with non-adherence to antiretroviral therapy and higher risk of AIDS and death: results from ACTG 362. AIDS care, 23(6), 775–785. https://doi.org/10.1080/09540121.2010.525617 (study 185)
74. Suonpera, E., Matthews, R., Milinkovic, A. et al. Risky Alcohol Consumption and Associated Health Behaviour Among HIV-Positive and HIV-Negative Patients in a UK Sexual Health and HIV Clinic: A Cross-Sectional Questionnaire Study. AIDS Behav 24, 1717–1726 (2020). https://doi.org/10.1007/s10461-019-02714-2
75. Tang, A.M., Hamunime, N., Adams, R.A. et al. Introduction of an Alcohol-Related Electronic Screening and Brief Intervention (eSBI) Program to Reduce Hazardous Alcohol Consumption in Namibia’s Antiretroviral Treatment (ART) Program. AIDS Behav 23, 3078–3092 (2019). https://doi.org/10.1007/s10461-019-02648-9
76. Kim, Ji & Yang, Youngran & Kim, Hyun. (2018). The Impact of Alcohol Use on Antiretroviral-Therapy Adherence in Koreans Living with HIV. Asian Nursing Research. 12. 10.1016/j.anr.2018.10.002.
77. Schensul JJ, Ha T, Schensul S, Sarna A, Bryant K. Identifying the Intersection of Alcohol, Adherence and Sex in HIV Positive Men on ART Treatment in India Using an Adapted Timeline Followback Procedure. AIDS Behav. 2017;21(Suppl 2):228-242. doi:10.1007/s10461-017-1916-1
78. Paolillo EW, Gongvatana A, Umlauf A, Letendre SL, Moore DJ. At-Risk Alcohol Use is Associated with Antiretroviral Treatment Nonadherence Among Adults Living with HIV/AIDS. Alcohol Clin Exp Res. 2017 Aug;41(8):1518-1525. doi: 10.1111/acer.13433. Epub 2017 Jul 5. PMID: 28679147; PMCID: PMC5564671.
79. Muessig KE, McLaughlin MM, Nie JM, Cai W, Zheng H, Yang L, Tucker JD. Suboptimal antiretroviral therapy adherence among HIV-infected adults in Guangzhou, China. AIDS Care. 2014;26(8):988-95. doi: 10.1080/09540121.2014.897912. Epub 2014 Mar 26. PMID: 24666239; PMCID: PMC4024070.
80. Denison JA, Koole O, Tsui S, et al. Incomplete adherence among treatment-experienced adults on antiretroviral therapy in Tanzania, Uganda and Zambia. AIDS. 2015;29(3):361-371. doi:10.1097/QAD.0000000000000543
81. Ferro EG, Weikum D, Vagenas P, et al. Alcohol use disorders negatively influence antiretroviral medication adherence among men who have sex with men in Peru. AIDS Care. 2015;27(1):93-104. doi:10.1080/09540121.2014.963013
82. Teixeira C, Dourado Mde L, Santos MP, Brites C. Impact of use of alcohol and illicit drugs by AIDS patients on adherence to antiretroviral therapy in Bahia, Brazil. AIDS Res Hum Retroviruses. 2013 May;29(5):799-804. doi: 10.1089/aid.2012.0296. Epub 2013 Feb 5. PMID: 23294471.
83. Peretti-Watel P, Spire B, Lert F, Obadia Y; VESPA Group. Drug use patterns and adherence to treatment among HIV-positive patients: evidence from a large sample of French outpatients (ANRS-EN12-VESPA 2003). Drug Alcohol Depend. 2006 Apr;82 Suppl 1:S71-9. doi: 10.1016/s0376-8716(06)80012-8. PMID: 16769450.
84. Roux P, Lions C, Cohen J, et al. Impact of HCV treatment and depressive symptoms on adherence to HAART among HIV-HCV-coinfected patients: results from the ANRS-CO13-HEPAVIH cohort. Antivir Ther. 2014;19(2):171-178. doi:10.3851/IMP2699
85. King RM, Vidrine DJ, Danysh HE, Fletcher FE, McCurdy S, Arduino RC, Gritz ER. Factors associated with nonadherence to antiretroviral therapy in HIV-positive smokers. AIDS Patient Care STDS. 2012 Aug;26(8):479-85. doi: 10.1089/apc.2012.0070. Epub 2012 May 21. PMID: 22612468; PMCID: PMC3407390.
86. Vieira-Castro ACM, Oliveira LCM. Impact of alcohol consumption among patients in hepatitis C virus treatment. Arq Gastroenterol. 2017 Jul-Sept;54(3):232-237. doi: 10.1590/S0004-2803.201700000-33. Epub 2017 Jul 13. PMID: 28724048.
87. Dever, J.B., Ducom, J.H., Ma, A. et al. Engagement in Care of High-Risk Hepatitis C Patients with Interferon-Free Direct-Acting Antiviral Therapies. Dig Dis Sci 62, 1472–1479 (2017). https://doi.org/10.1007/s10620-017-4548-4
88. Wagner GJ, Ghosh-Dastidar B, Mukasa B, Linnemayr S. Changes in ART Adherence Relate to Changes in depression as Well! Evidence for the Bi-directional Longitudinal Relationship Between Depression and ART Adherence from a Prospective Study of HIV Clients in Uganda. AIDS Behav. 2020 Jun;24(6):1816-1824. doi: 10.1007/s10461-019-02754-8. PMID: 31813077; PMCID: PMC7228829.
89. Culbert, G. J., Waluyo, A., Wang, M., Putri, T. A., Bazazi, A. R., & Altice, F. L. (2019). Adherence to Antiretroviral Therapy Among Incarcerated Persons with HIV: Associations with Methadone and Perceived Safety. AIDS and behavior, 23(8), 2048–2058. https://doi.org/10.1007/s10461-018-2344-6 (study 9)
90. Cunningham, E. B., Hajarizadeh, B., Amin, J., Litwin, A. H., Gane, E., Cooper, C., Lacombe, K., Hellard, M., Read, P., Powis, J., Dalgard, O., Bruneau, J., Matthews, G. V., Feld, J. J., Dillon, J. F., Shaw, D., Bruggmann, P., Conway, B., Fraser, C., Marks, P., … SIMPLIFY and D3FEAT study groups (2020). Adherence to Once-daily and Twice-daily Direct-acting Antiviral Therapy for Hepatitis C Infection Among People With Recent Injection Drug Use or Current Opioid Agonist Therapy. Clinical infectious diseases : an official publication of the Infectious Diseases Society of America, 71(7), e115–e124. https://doi.org/10.1093/cid/ciz1089 (study 139)
91. Ingersoll K. (2004). The impact of psychiatric symptoms, drug use, and medication regimen on non-adherence to HIV treatment. AIDS care, 16(2), 199–211. https://doi.org/10.1080/09540120410001641048 (study 224)
92. Lambers, F. A., Stolte, I. G., van den Berg, C. H., Coutinho, R. A., & Prins, M. (2011). Harm reduction intensity-Its role in HAART adherence amongst drug users in Amsterdam. The International journal on drug policy, 22(3), 210–218. https://doi.org/10.1016/j.drugpo.2011.02.004 (study 55)
93. Mimiaga, M. J., Reisner, S. L., Grasso, C., Crane, H. M., Safren, S. A., Kitahata, M. M., Schumacher, J. E., Mathews, W. C., & Mayer, K. H. (2013). Substance use among HIV-infected patients engaged in primary care in the United States: findings from the Centers for AIDS Research Network of Integrated Clinical Systems cohort. American journal of public health, 103(8), 1457–1467. https://doi.org/10.2105/AJPH.2012.301162 (study 117)
94. Nolan, S., Walley, A. Y., Heeren, T. C., Patts, G. J., Ventura, A. S., Sullivan, M. M., Samet, J. H., & Saitz, R. (2017). HIV-infected individuals who use alcohol and other drugs, and virologic suppression. AIDS care, 29(9), 1129–1136. https://doi.org/10.1080/09540121.2017.1327646 (study 65)
95. Palepu, A., Tyndall, M. W., Joy, R., Kerr, T., Wood, E., Press, N., Hogg, R. S., & Montaner, J. S. (2006). Antiretroviral adherence and HIV treatment outcomes among HIV/HCV co-infected injection drug users: the role of methadone maintenance therapy. Drug and alcohol dependence, 84(2), 188–194. https://doi.org/10.1016/j.drugalcdep.2006.02.003 (study 12)
96. Paschen-Wolff, M. M., Campbell, A., Tross, S., Choo, T. H., Pavlicova, M., & Jarlais, D. D. (2020). DSM-5 substance use disorder symptom clusters and HIV antiretroviral therapy (ART) adherence. AIDS care, 32(5), 645–650. https://doi.org/10.1080/09540121.2019.1686600 (study 152)
97. Shannon, K., Kerr, T., Lai, C., Ishida, T., Wood, E., Montaner, J. S., Hogg, R. S., & Tyndall, M. W. (2005). Nonadherence to antiretroviral therapy among a community with endemic rates of injection drug use. Journal of the International Association of Physicians in AIDS Care (Chicago, Ill. : 2002), 4(3), 66–72. https://doi.org/10.1177/1545109705284353 (study 210)
98. De Boni RB, Shepherd BE, Grinsztejn B, Cesar C, Cortés C, Padgett D, Gotuzzo E, Belaunzarán-Zamudio PF, Rebeiro PF, Duda SN, McGowan CC. Substance Use and Adherence Among People Living with HIV/AIDS Receiving cART in Latin America. AIDS Behav. 2016 Nov;20(11):2692-2699. doi: 10.1007/s10461-016-1398-6. PMID: 27091028; PMCID: PMC5069110.
99. Roux P, Lions C, Cohen J, et al. Impact of HCV treatment and depressive symptoms on adherence to HAART among HIV-HCV-coinfected patients: results from the ANRS-CO13-HEPAVIH cohort. Antivir Ther. 2014;19(2):171-178. doi:10.3851/IMP2699
100. King RM, Vidrine DJ, Danysh HE, Fletcher FE, McCurdy S, Arduino RC, Gritz ER. Factors associated with nonadherence to antiretroviral therapy in HIV-positive smokers. AIDS Patient Care STDS. 2012 Aug;26(8):479-85. doi: 10.1089/apc.2012.0070. Epub 2012 May 21. PMID: 22612468; PMCID: PMC3407390.
101. Hinkin CH, Barclay TR, Castellon SA, Levine AJ, Durvasula RS, Marion SD, Myers HF, Longshore D. Drug use and medication adherence among HIV-1 infected individuals. AIDS Behav. 2007 Mar;11(2):185-94. doi: 10.1007/s10461-006-9152-0. PMID: 16897351; PMCID: PMC2867605.
102. Cohen MH, Cook JA, Grey D, et al. Medically eligible women who do not use HAART: the importance of abuse, drug use, and race. Am J Public Health. 2004;94(7):1147-1151. doi:10.2105/ajph.94.7.1147
103. Ingersoll K. The impact of psychiatric symptoms, drug use, and medication regimen on non-adherence to HIV treatment. AIDS Care. 2004 Feb;16(2):199-211. doi: 10.1080/09540120410001641048. PMID: 14676026.
104. Tucker JS, Burnam MA, Sherbourne CD, Kung FY, Gifford AL. Substance use and mental health correlates of nonadherence to antiretroviral medications in a sample of patients with human immunodeficiency virus infection. Am J Med. 2003 May;114(7):573-80. doi: 10.1016/s0002-9343(03)00093-7. PMID: 12753881.
105. Lucas GM, Gebo KA, Chaisson RE, Moore RD. Longitudinal assessment of the effects of drug and alcohol abuse on HIV-1 treatment outcomes in an urban clinic. AIDS. 2002 Mar 29;16(5):767-74. doi: 10.1097/00002030-200203290-00012. PMID: 11964533.
106. Barkham, M., Stiles, W.B., Connell, J., Twigg, E., Leach, C., Lucock, M., Mellor-Clark, J., Bower, P., King, M., Shapiro, D.A., Hardy, G.E., Greenberg, L.S., & Angus, L. Effects of psychological therapies in randomized trials and practice-based studies. British J Clin Psych. 2008 Nov; 47 (4), 397-415. doi: 10.1348/014466508X311713. Epub 2008 Jul 12. PMID: 18625084.
107. Lane C, McCrabb S, Nathan N, Naylor PJ, Bauman A, Milat A, Lum M, Sutherland R, Byaruhanga J, Wolfenden L. How effective are physical activity interventions when they are scaled-up: a systematic review. Int J Behav Nutr Phys Act. 2021 Jan 22;18(1):16. doi: 10.1186/s12966-021-01080-4. PMID: 33482837; PMCID: PMC7821550.
